# PersonALL: a genetic scoring guide for personalized risk assessment in pediatric B-cell precursor acute lymphoblastic leukemia

**DOI:** 10.1038/s41416-023-02309-8

**Published:** 2023-06-21

**Authors:** Gábor Bedics, Bálint Egyed, Lili Kotmayer, Anne Benard-Slagter, Karel de Groot, Anna Bekő, Lajos László Hegyi, Bence Bátai, Szilvia Krizsán, Gergely Kriván, Dániel J. Erdélyi, Judit Müller, Irén Haltrich, Béla Kajtár, László Pajor, Ágnes Vojcek, Gábor Ottóffy, Anikó Ujfalusi, István Szegedi, Lilla Györgyi Tiszlavicz, Katalin Bartyik, Krisztina Csanádi, György Péter, Réka Simon, Péter Hauser, Ágnes Kelemen, Endre Sebestyén, Zsuzsanna Jakab, András Matolcsy, Csongor Kiss, Gábor Kovács, Suvi Savola, Csaba Bödör, Donát Alpár

**Affiliations:** 1 HCEMM-SE Molecular Oncohematology Research Group, Department of Pathology and Experimental Cancer Research, Semmelweis University, Budapest, Hungary; 2 Department of Pediatrics, Semmelweis University, Budapest, Hungary; 3 MRC Holland, Amsterdam, The Netherlands; 4 Central Hospital of Southern Pest - National Institute of Hematology and Infectious Diseases, Budapest, Hungary; 5 Department of Pathology, University of Pécs Medical School, Pécs, Hungary; 6 Department of Pediatrics, University of Pécs Medical School, Pécs, Hungary; 7 Department of Laboratory Medicine, Faculty of Medicine, University of Debrecen, Debrecen, Hungary; 8 Division of Pediatric Hematology-Oncology, Institute of Pediatrics, Faculty of Medicine, University of Debrecen, Debrecen, Hungary; 9 Department of Paediatrics and Paediatric Health Care Center, Faculty of Medicine, University of Szeged, Szeged, Hungary; 10 Hemato-Oncology Unit, Heim Pál Children’s Hospital, Budapest, Hungary; 11 Hemato-Oncology and Stem Cell Transplantation Unit, Velkey László Children’s Health Center, Miskolc, Hungary; 12 Hungarian Childhood Cancer Registry, Hungarian Pediatric Oncology Network, Budapest, Hungary; 13 Department of Laboratory Medicine, Karolinska Institute, Solna, Sweden

**Keywords:** Acute lymphocytic leukaemia, Cancer genetics, Prognostic markers

## Abstract

**Background:**

Recurrent genetic lesions provide basis for risk assessment in pediatric acute lymphoblastic leukemia (ALL). However, current prognostic classifiers rely on a limited number of predefined sets of alterations.

**Methods:**

Disease-relevant copy number aberrations (CNAs) were screened genome-wide in 260 children with B-cell precursor ALL. Results were integrated with cytogenetic data to improve risk assessment.

**Results:**

CNAs were detected in 93.8% (*n* = 244) of the patients. First, cytogenetic profiles were combined with *IKZF1* status (*IKZF1*^normal^, *IKZF1*^del^ and *IKZF1*^plus^) and three prognostic subgroups were distinguished with significantly different 5-year event-free survival (EFS) rates, IKAROS-low (*n* = 215): 86.3%, IKAROS-medium (*n* = 27): 57.4% and IKAROS-high (*n* = 18): 37.5%. Second, contribution of genetic aberrations to the clinical outcome was assessed and an aberration-specific score was assigned to each prognostically relevant alteration. By aggregating the scores of aberrations emerging in individual patients, personalized cumulative values were calculated and used for defining four prognostic subgroups with distinct clinical outcomes. Two favorable subgroups included 60% of patients (*n* = 157) with a 5-year EFS of 96.3% (excellent risk, *n* = 105) and 87.2% (good risk, *n* = 52), respectively; while 40% of patients (*n* = 103) showed high (*n* = 74) or ultra-poor (*n* = 29) risk profile (5-year EFS: 67.4% and 39.0%, respectively).

**Conclusions:**

PersonALL, our conceptually novel prognostic classifier considers all combinations of co-segregating genetic alterations, providing a highly personalized patient stratification.

## Introduction

Cancer is a leading cause of death among children in the Western countries, with leukemia being the most common malignant disorder in this age group [1]. Acute lymphoblastic leukemia (ALL) accounts for 80% of all pediatric leukemia cases and displays B-cell precursor phenotype (B-ALL) in approximately 85% of the patients [2]. Pediatric ALL develops in multiple steps, with the initiating genomic lesion emerging in utero, as demonstrated in major genetic subtypes, followed by the rise of secondary aberrations required for the clinical manifestation of the disease [3–5]. Copy number aberrations (CNAs) such as whole chromosome gains and losses as well as subchromosomal imbalances recurrently occur as primary or secondary alterations, substantially contributing to the heterogeneous genomic landscape of ALL [6–9].

The vast majority of numerical chromosome aberrations emerge in the high-hyperdiploid subgroup which accounts for 25–30% of pediatric B-ALL patients, with the leukemic blasts in this subgroup bearing non-random gains of specific chromosomes conferring a modal chromosome number of 51-67 [10, 11]. Subchromosomal CNAs recurrently affect genes involved in cell cycle control, tumor suppression, lymphoid cell development and B-cell differentiation [6]. A wide range of methods is available for the screening of CNAs in pediatric ALL, including karyotyping, fluorescence in situ hybridization (FISH), DNA index measurement, multiplex ligation-dependent probe amplification (MLPA), cytogenomics, optical genome mapping as well as various array- and next-generation sequencing (NGS) based approaches [5, 6, 12–14]. digitalMLPA is a recently developed technique which combines MLPA with NGS readout providing a high-throughput, scalable, highly rationalized but still comprehensive means to interrogate recurrently affected genomic loci with a short turn-around time as previously demonstrated by our group and others [15–17].

Several studies investigated the clinical significance of CNAs in pediatric ALL and identified a range of prognostic biomarkers based on modal chromosome number [18–21], specific trisomies [19, 20, 22, 23], double or triple trisomies [19–21, 24, 25], simultaneous presence and absence of various trisomies [26], loss or gain/amplification of key driver genes [27, 28], as well as specific alteration patterns of predefined gene sets [29]. These observations facilitated the widespread implementation of CNA screening in the diagnostics of pediatric ALL, with an aim to support patient stratification and potentially aid therapy selection. Integrative efforts have led to the establishment of complex classifiers enabling the assignment of patients to distinct prognostic subgroups based on cytogenetic and molecular genetic markers [16, 29–31]. Shortcomings of current genetic classifiers are the relatively low number and limited combinations of aberrations used as criteria for decision making. Assignment of individual patients is typically restricted to a couple of specific genomic patterns; for example, trisomy of chromosomes 17 and/or 18 without extra copies of chromosomes 5 and 20; isolated *IKZF1* deletion; isolated deletion of *ETV6*, *PAX5* or *BTG1*; co-occurrence of *IKZF1* deletion with deletion of *CDKN2A*, *CDKN2B*, *PAX5* or PAR1 in the absence of *ERG* deletion; *ETV6* deletion with single deletion of *BTG1*, *CDKN2A/B* or *PAX5*, with all other uncategorizable patients being classified in the same non-specific, collective subgroup.

In this study, we performed a comprehensive screening for disease-relevant CNAs in a cohort of Hungarian patients diagnosed with pediatric B-ALL using digitalMLPA. The generated CNA profiles were combined with cytogenetic data for risk assessment. Besides integrating *IKZF1* status (*IKZF1*^normal^, *IKZF1*^del^ and *IKZF1*^plus^) with cytogenetic classes, thus creating a cytogenetics aware interpretation of *IKZF1* imbalance, we introduced a conceptually novel patient classification approach called PersonALL, which assigns patients to prognostic subgroups based on highly individualized cumulative scores reflecting the weighted impact of all relevant aberrations detected in a particular patient. This newly developed prognostic classifier which flexibly considers all possible combinations of screened and potentially co-segregating genetic alterations provides a more refined, hence more personalized risk assessment for children with B-ALL.

## Materials and methods

### Patient samples

In the frame of the Hungarian Pediatric Leukemia Molecular Profiling Program, diagnostic bone marrow samples from 260 patients (male:female ratio: 1.43:1) diagnosed with B-cell precursor ALL at age 1–17 years (median: 5 years) were investigated (Table S1). Diagnoses were made based on morphological, immunophenotypical and genotypical criteria in the Department of Pathology and Experimental Cancer Research, Semmelweis University, in the Department of Pathology, University of Pécs, or in the Department of Pathology, University of Debrecen between 2003 and 2019 [2, 32]. Specimens contained on average 79% (range: 29–99%) leukemic blasts as assessed by flow cytometry (Table S1). Baseline genetic characterization of patient samples included DNA index measurement by flow cytometry, karyotyping by GTG-banding, FISH for *BCR-ABL1* and *ETV6-RUNX1* fusions, *KMT2A* and *E2A* rearrangements, and trisomy of chromosomes 4 and 10 using Vysis probes (Abbott Diagnostics, Abbott Park, IL, USA), as well as quantitative PCR tests for *BCR-ABL1* and *ETV6-RUNX1* fusions, and conventional MLPA using the SALSA 202 and 335 probemixes (MRC-Holland, Amsterdam, the Netherlands). Risk assessment and treatment selection were performed according to ALL IC-BFM protocols, such as the ALL IC-BFM 2002 (121 patients, 47%) and ALL IC-BFM 2009 (139 patients, 53%) (Table S1) [33]. Standard-risk (SR), medium-risk (MR) and high-risk (HR) groups were represented by 35%, 52% and 13% as well as by 16%, 65% and 19% of the patients in cohorts treated with ALL IC-BFM 2002 and ALL IC-BFM 2009, respectively. The estimated 5-year event-free survival (EFS) rates were similar in groups of patients treated with different versions of the ALL IC-BFM protocol (ALL IC-BFM 2002: 77.8%, ALL IC-BFM 2009: 81.6%, log-rank test: *p* = 0.530, Fig. S1); therefore, data from all 260 patients were combined. In the entire discovery cohort, 65 (25%), 153 (59%) and 42 (16%) patients were stratified in the SR, MR and HR groups, respectively. Ethical approval (45563-2/2019/EKU) from the Medical Research Council of Hungary and written informed consent from the patients and/or from the parents or guardians were obtained for the study, which was conducted in accordance with the Declaration of Helsinki.

### digitalMLPA

digitalMLPA reactions were performed on 40 ng genomic DNA using the D007 ALL probemix (version D007-X2-0516 or D007-X5-0220), which was developed by the MRC-Holland and provided to collaborating laboratories for testing and validation. The probemix consisted of (*i*) target probes for regions recurrently altered by copy number aberrations in acute lymphoblastic leukemia; (*ii*) digital karyotyping probes covering all chromosome arms for detection of gross chromosomal aberrations and serving as reference probes for data normalization, and (*iii*) internal control probes for quality control and sample identification. List and position of probes included in the D007-X2-0516 and D007-X5-0220 versions of the D007 probemix are presented in Table S2.

digitalMLPA reactions were carried out according to the previously published protocol [15, 16]. Briefly, individual DNA samples were mixed with a unique barcode solution followed by sample denaturation and addition of digitalMLPA probes with digitalMLPA buffer to the reaction mix. Each probe comprised two oligonucleotides with a locus-specific 25–50 bp hybridizing sequence. Probe oligonucleotides binding to target region were designed to hybridize adjacently; hence, if perfectly bound, could be ligated into a complete probe using the ligase-65 enzyme. Ligated probes were amplified by universal primers compatible with Illumina sequencing platforms. Sample-specific PCR products from different reactions were pooled and sequenced on a MiSeq v3 standard flow cell (Illumina, San Diego, CA, USA) using 110 bp or 115 bp single-read chemistry.

Copy number status at each interrogated locus was determined from the NGS output in two consecutive steps using the Coffalyser digitalMLPA software v.004 (MRC Holland). First, read count for each probe was normalized by the read counts generated from reference probes hybridizing to copy number stable regions of the same genome. Second, the relative read count calculated for each probe was compared with the matching values of all reference samples. The final probe ratio value (dosage quotient) was around 1.0 if the analyzed region was unaffected by CNA, while an increased or decreased value indicated the presence and level of gain or loss, respectively. Leukemic cell purity as assessed by flow cytometry was also considered at the interpretation of the results. CNAs were reported as being subclonal if multiple consecutive probes had dosage quotients unambiguously falling outside the normal range but without reaching the expected level of monoallelic loss as calculated based on sample purity, and also compared with other altered regions within the same specimen. Detailed laboratory and bioinformatic protocols as well as validation methods have previously been published [15, 16].

### Validation cohort—origin and analysis

The independent validation cohort comprised 606 patients included in the Acute Lymphoblastic Leukemia Pilot Phase 1 (phs0000463) or the Expansion Phase 2 (phs000464) studies of the Therapeutically Applicable Research to Generate Effective Treatments—TARGET initiative (https://ocg.cancer.gov/programs/target), and genomically profiled at the St. Jude Children’s Research Hospital or at the Baylor College of Medicine using Gene Chip Human Mapping 500 K Array (Affymetrix) or SeqCap EZ Human Exome 2.0 (Nimblegen), respectively (Table S3). Data used for the analysis is available at https://portal.gdc.cancer.gov/projects. Patients diagnosed with B-ALL at age <18 and having reported EFS values were included, while exclusion criteria comprised Down-syndrome and early toxicity during induction therapy. After reviewing the Affymetrix 500 K Array results downloaded from the TARGET website, CNAs with copy number segments of >2.3 or <0.7 as reported by the St. Jude Children’s Research Hospital were considered in our validation analyses. Whole-exome sequencing derived BAM files aligned to the reference genome Human Build 37 (NCBI) were downloaded and CNAs were called by the CNVkit v.0.9.10.dev0 utilizing a circular binary segmentation method [34–36]. Log2 ratio estimates were normalized based on sex of the patients and on reported leukemic cell purity assessed by flow cytometry. Genetic subgroups and 5-year EFS in the validation cohort and in our in-house discovery cohort are shown in Table S4 and Fig. S2.

### Statistical analysis

Co-segregation of disease-relevant CNAs were analyzed using the “somaticInteraction” function of maftools Bioconductor package (v2.2.10) which performed pairwise Fisher’s exact tests and identified significant mutually exclusive or co-occurring events [37]. Event-free survival (EFS) up to 5 years was defined as the time from start of treatment to relapse, second malignancy or disease-related death, excluding early toxicity. Mean follow-up time was 46.6 months (range: 1.5–72.0 months) with at least 24.0 months at patients experiencing no event during the study period. Cox regression models were used for assessing the association of individual genetic aberrations with risk of progression and for building models for progression prediction. Survival rates were estimated using the Kaplan-Meier method and compared by log-rank tests coupled with Benjamini-Hochberg false discovery rate correction. Statistical analyses were performed using R version 4.1.2 (R Foundation for Statistical Computing, Vienna, Austria, 2021).

## Results

### Frequency and distribution of chromosomal and subchromosomal copy number aberrations

In total, 1398 CNAs including gross chromosomal alterations and subchromosomal lesions were detected in 244/260 (93.8%) diagnostic patient samples. On average, 5.4 CNAs were observed per patient with a mean of 2.5 subchromosomal aberrations. Ninety percent of whole chromosome changes were observed in patients bearing hyperdiploid karyotype with 4–14 affected chromosomes, predominantly extra copies of chromosomes 21, 6, X, 14, 18, 17, 4 and 10 (Fig. S3). Gain of multiple copies was recurrently observed at chromosomes 21, X, 14 and 18. Modal chromosome number among the 82 patients harboring high-hyperdiploid karyotype ranged between 51 and 62 with a median of 55, while one patient displayed near-triploidy with 72 chromosomes.

Subchromosomal CNAs were identified in 208/260 (80.0%) patients with *VPREB1* deletion being the most common lesion occurring in 32.3% of the cases. Additional genes altered with a frequency of at least 5% in our patient cohort included various cell cycle control, lymphoid development, signaling or tumor suppressor genes such as *CDKN2A/B*, *ETV6*, *PAX5*, *IKZF1*, *MLLT3*, *TBL1XR1*, *BTG1, RB1, BTLA/CD200, CASP8AP2, RUNX1* as well as the PAR1 region (Table 1). Seventy percent of biallelic losses included the *VPREB1* and *CDKN2A/B* genes while over two-thirds of the multiple gains affected *RUNX1*. Approximately 8% of the subchromosomal CNAs were detected as subclonal aberrations which encompassed the *ETV6*, *PAX5*, *CDKN2A/B*, *IKZF1* and *VPREB1* genes in majority of the cases. Dosage quotient values indicating the fusion and amplification of the *NUP214* and *ABL1* genes were observed in two patients. Considering the genetic subtypes of pediatric B-ALL, the highest average numbers of subchromosomal aberrations (4.8–5.0 CNAs per patient) were observed in the *BCR-ABL1*-positive and iAMP21 subgroups, while the lowest values with 1.2–1.7 CNAs per patient were associated with hyperdiploidy and *TCF3-PBX1* fusion (Table S5).

**Table 1:** Targets of subchromosomal copy number aberrations detected in diagnostic samples of our 260 patients and ranked in order of frequency.

Lesion	Gene	Total number of aberrations	Biallelic losses / multiple gains	Subclonal alterations
Loss	*VPREB1*	84	18	5
	*CDKN2A/B*	72	28	5
	*ETV6*	68	4	6
	*PAX5*	43	0	6
	*IKZF1*	34	1	5
	*MLLT3*	28	5	4
	*PAR1*	20	0	2
	*TBL1XR1*	19	1	0
	*BTG1*	17	0	0
	*RB1*	15	6	0
	*BTLA/CD200*	15	0	0
	*CASP8AP2*	15	0	2
	*TP53*	10	1	1
	*CTCF*	9	0	1
	*NR3C1*	8	0	2
	*EBF1*	5	0	0
	*ERG*	4	0	0
	*LEF1*	4	0	0
	*PTEN*	4	0	2
	*NF1*	3	0	0
	*NR3C2*	3	0	0
	*EZH2*	2	0	0
	*SUZ12*	2	0	0
	*IKZF2*	1	1	0
	*NOTCH1*	1	0	0
	*PHF6*	1	0	0
	*PTPN2*	1	0	0
Gain	*RUNX1*	23	11	1
	*PHF6*	11	4	0
	*ABL1*	6	1	0
	*MYB*	1	0	0

**Fig. 1:**
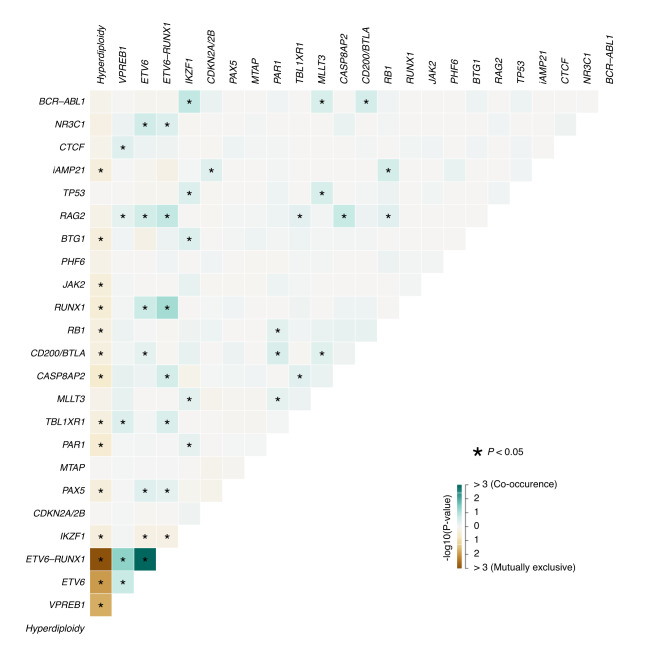
Co-segregation analysis of genetic subgroup defining alterations and copy number aberrations affecting genes recurrently altered in pediatric B-ALL. Co-occurrence and mutual exclusivity between various B-ALL subgroup defining alterations and/or detected copy number aberrations are labeled with green and brown colors, respectively. Significant (*p* < 0.05) associations revealed by pair-wise Fisher’s exact test are marked with asterisks.

Simultaneous presence of various CNAs and B-ALL subgroup defining alterations was investigated in order to reveal potential associations between individual genetic lesions (Fig. 1). The vast majority of mutual exclusivity or negative correlations was observed in the high-hyperdiploid subgroup, while the pairwise analyses revealed 35 significant positive correlations across various disease subtypes. The strongest positive associations were observed in the *ETV6-RUNX1* subgroup and included *ETV6* loss, *RUNX1* gain and *VPREB1* loss. Among patients with iAMP21, enrichment of *CDKN2A/B* loss and *RB1* deletion was observed. *IKZF1*, *MLLT3* and *CD200/BTLA* losses were associated with *BCR-ABL1* positivity, while *IKZF1* loss showed negative correlation with *ETV6-RUNX1* fusion and hyperdiploidy. Beyond that, *IKZF1* deletion showed significant co-occurrence with *TP53*, *BTG1* and *MLLT3* as well as with deletion of the PAR1 region.

### Hyperdiploidy and prognosis

**Fig. 2:**
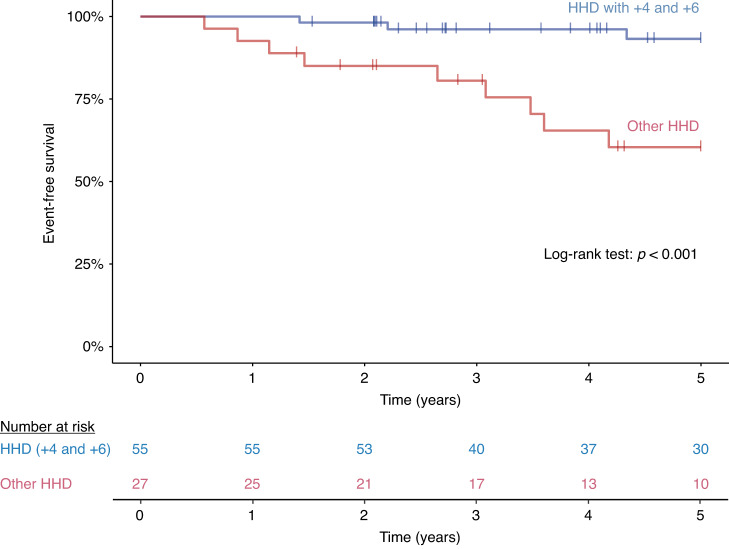
Event-free survival of high-hyperdiploid patients with presence or absence of double trisomy affecting chromosomes 4 and 6. High-hyperdiploid patients with extra chromosomes of 4 and 6 showed higher estimated 5-year EFS rate than other high-hyperdiploid patients in our cohort (93.2% vs. 60.4%).

Whole chromosome gains were investigated individually and in combinations to identify specific patterns associated with favorable outcome among patients with high-hyperdiploid karyotype as revealed or confirmed by digitalMLPA. Simultaneous survival rate analyses using the Kaplan-Meier method unraveled multiple single, double and triple trisomies as markers of good risk, mainly including various combinations of chromosomes 4, 6, 10, 17 and 18, and excluding gains of chromosomes 5 and 20. Due to multiple statistical comparisons of these combinations, results were corrected using the Bonferroni method, after which double trisomy of chromosomes 4 and 6 remained as the only marker significantly associated with superior outcome within the high-hyperdiploid subgroup (Fig. 2).

### *IKZF1* status and its prognostic value

**Fig. 3:**
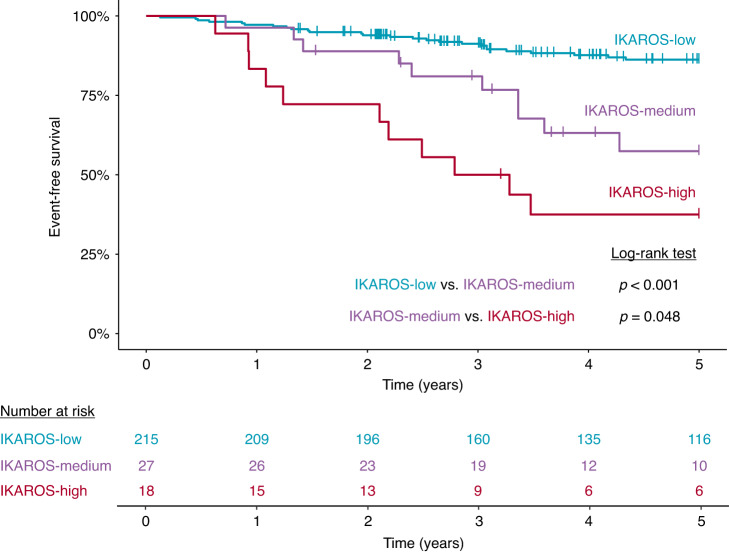
Event-free survival of 260 patients classified by combined cytogenetic and *IKZF1* copy number status. Risk groups were determined as outlined in Fig. S6. Patients in the IKAROS-low, IKAROS-medium and IKAROS-high groups showed an estimated 5-year EFS rate of 86.3%, 57.4% and 37.5%, respectively.

The D007 probemix covers all exons of the *IKZF1* gene with two probes, as well as regions located approximately 4 and 2 kilobases upstream of the coding sequences, enabling a fine mapping of deletions affecting the *IKZF1* gene. In patients harboring *IKZF1* loss, 10 different patterns of deletion were observed, predominantly exons 4–7 and exons 1–7 losses, as well as deletion of the whole gene including the upstream region (Fig. S4). Notably, we observed a non-random distribution of *IKZF1*^del^ and *IKZF1*^plus^ statuses across patients displaying different patterns of *IKZF1* deletion. Eight out of nine patients with exons 4–7 loss and 6/7 patients with upstream region/exons 1–8 (i.e. whole *IKZF1*) deletion showed *IKZF1*^plus^ CNA profile. On the other hand, all patients with exon 1–7 deletion belonged to the *IKZF1*^del^ group, without meeting the criteria of *IKZF1*^plus^. By analyzing the prognostic value of *IKZF1* status in our patient cohort, a decreasing rate of EFS was observed in patents with normal vs *IKZF1*^del^ vs *IKZF1*^plus^ genotype; however, the difference between the latter two categories did not reach statistical significance (Fig. S5). We hypothesized that co-evaluation of *IKZF1* status and additional cytogenetic features interdependently may improve the *IKZF1*-driven prognostic risk assessment. In order to generate these novel subgroups, we combined the *IKZF1* status with cytogenetic categories defined and applied successfully in previous studies (Fig. S6) [16, 30, 31], which eventually allowed for distinguishing three prognostic groups (IKAROS-low, IKAROS-medium and IKAROS-high) with significantly different 5-year EFS (Fig. 3).

### Integrative genetic classification for personalized risk assessment

**Fig. 4:**
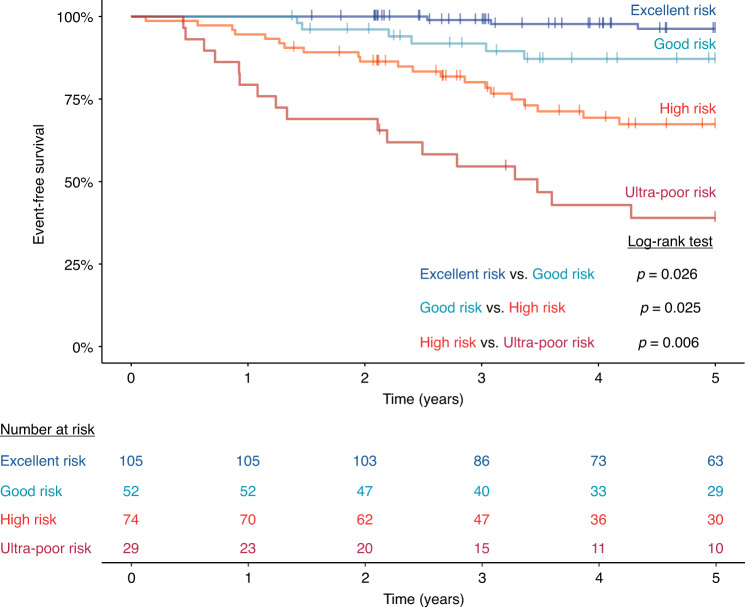
Event-free survival of 260 patients with B-cell precursor ALL, classified according to patient-specific composition of all disease-relevant aberrations associated with prognostic significance in our patient cohort. Excellent, good, high and ultra-poor risk groups showed an estimated 5-year EFS rate of 96.3%, 87.2%, 67.4% and 39.0%, respectively. Patient-specific scores were calculated as outlined in Table S6.

**Fig. 5:**
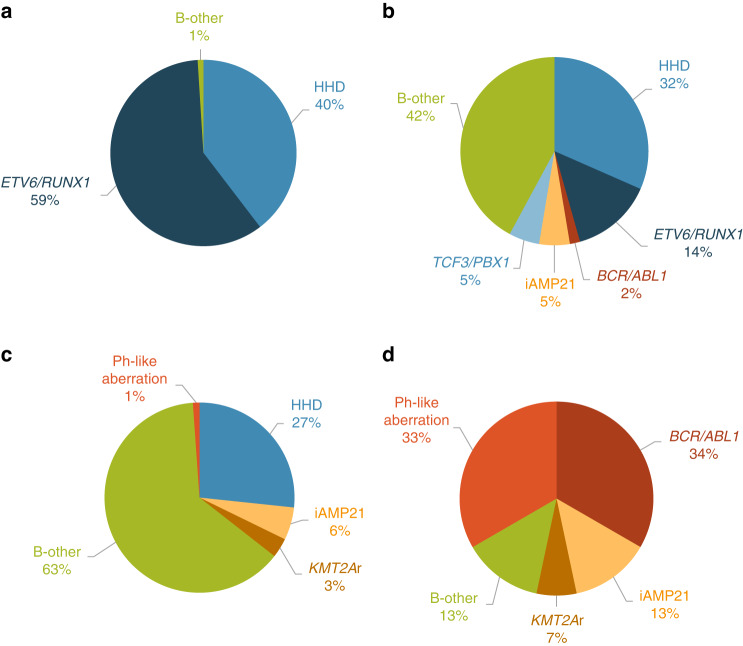
Distribution of cytogenetic subtypes across four risk groups determined based on non-overlapping ranges of patient specific cumulative scores generated from the prognostic value of single genetic lesions. **a** The excellent risk group almost exclusively comprised patients with *ETV6/RUNX1* gene fusion or high-hyperdiploid (HHD) karyotype. **b**, **c** The good and the high-risk groups were dominated by patients who classified as B-other in the current study. **d** Two-thirds of the patients in the ultra-poor risk group carried *BCR/ABL1* fusion or alterations characteristic of the Ph-like subtype, with an additional one-fifth of the children in this group harboring *KMT2A* rearrangement or iAMP21 genotype.

In order to establish a highly personalized risk assessment of patients with B-cell precursor ALL, we introduced a novel classification called PersonALL, which flexibly takes account of the unique composition of aberrations in individual patients. First, prognostic significance of all disease-relevant, recurrent genetic aberrations detected by digitalMLPA or by conventional approaches such as karyotyping and FISH was evaluated in our patient cohort using univariate Cox proportional hazard models. Second, aberrations with a frequency of >1.5% and a Cox model hazard ratio of >1.5 or ≤0.66 were selected and used for calculating patient-specific cumulative scores. The scoring system proportionally weighted individual cytogenetic aberrations and subchromosomal CNAs as outlined in Table S6. Patient-specific cumulative scores generated from the prognostic value of single genetic lesions distinguished four prognostic subgroups with excellent (score: ≥4), good (score: 0–3), high (score: –1 to –4) and ultra-poor risk (score: ≤ –4), demonstrating significantly different 5-year EFS rates (Fig. 4). The excellent, good, high and ultra-poor risk groups comprised 40.4%, 20.0%, 28.5% and 11.1% of the patients, respectively. The excellent risk group almost exclusively contained patients with *ETV6/RUNX1* fusion or high-hyperdiploid karyotype with common double trisomy of chromosomes 4 and 6. An increased fraction of B-other cases coupled with reduced representativity of *ETV6/RUNX1* positivity and high-hyperdiploidy was observed in the good risk group. In the high-risk group, almost two-thirds of the patients were classified as B-other, while the ultra-poor risk group was enriched for *BCR/ABL1* gene fusion, other gene fusions including *CRLF2* and *ABL* class aberrations characteristic of Ph-like signature and iAMP21 genotype (Fig. 5).

**Fig. 6:**
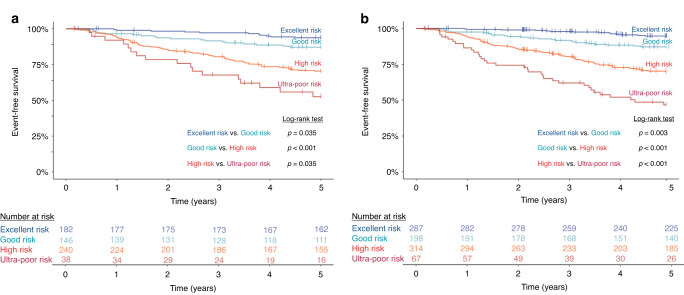
Validation of PersonALL, a novel classification approach assigning patients to four prognostic subgroups based on highly individualized cumulative scores reflecting the weighted impact of all detected and prognostically relevant genetic aberrations. **a** Event-free survival of 606 patients with B-cell precursor ALL included in the TARGET ALL Phase 1 Pilot or Phase 2 Expansion studies. Excellent, good, high and ultra-poor risk groups showed an estimated 5-year EFS rate of 93.8%, 87.2%, 70.4% and 52.5%, respectively. **b** Event-free survival of 866 (260 + 606) patients with B-cell precursor ALL included in the merged discovery (in-house) and validation cohort. Excellent, good, high and ultra-poor risk groups showed an estimated 5-year EFS rate of 94.7%, 87.2%, 69.8% and 46.8%, respectively.

Performance of PersonALL was validated on an independent cohort of 606 patients included in the TARGET ALL Phase 1 Pilot and Phase 2 Expansion studies, using a scoring scheme identical to the one applied at our in-house discovery cohort. Comparison of the excellent, good, high and ultra-poor risk groups consisting of 30.0%, 24.1%, 39.6% and 6.3% of the patients, respectively, demonstrated significantly different 5-year EFS rates (Fig. 6a). In addition, we tested this novel risk assessment method on the merged dataset comprising relevant information from all patients (*n* = 866) included in the validation cohort and in our discovery cohort. The difference in 5-year EFS across the risk groups showed even higher statistical significance than observed in the validation cohort, thus providing further confirmation on the value and robustness of our newly introduced prognostic classifier (Fig. 6b).

## Discussion

Risk assessment based on molecular features of leukemic blasts is gaining increased importance in the clinical stratification of children with B-ALL. Several recurrent CNAs including whole chromosome aberrations and focal alterations have prognostic and/or predictive significance which has led to the incorporation of CNAs into various risk classifiers [16, 26, 29–31]. While co-segregation of different driver aberrations was reported in large-scale genomic studies, flexible prognostic classification approaches adaptively considering the specific combination of genetic lesions in individual patients have not been established to date.

In this study, we used digitalMLPA, a robust high-throughput method for screening disease-relevant CNAs in a nation-wide cohort of Hungarian children diagnosed with B-ALL. While the general feasibility of our approach had previously been demonstrated [16], the significantly larger patient population in the current analysis allowed us to go beyond a simple validation of observations made before. Detection of chromosomal and large subchromosomal CNAs along with exon-level mapping of focal driver aberrations provided prognostically relevant results by (*i*) unveiling chromosomal gains associated with the most favorable EFS in the high-hyperdiploid subgroup, (*ii*) identifying *IKZF1* deletion patterns specifically associated with *IKZF1*^del^ and *IKZF1*^plus^ genotypes, (*iii*) facilitating the establishment of a cytogenetically aware patient classification based on IKAROS status, and most importantly, (*iv*) allowing us to design and successfully test a conceptually novel prognostic classifier called PersonALL which dynamically takes into account all possible combinations of potentially co-segregating genetic aberrations screened by digitalMLPA and/or more conventional methods.

Molecular and cytogenetic characterization of the diagnostic samples provided evidence of representativity of our patient cohort in terms of genetic subgroups, frequency of chromosomal and subchromosomal driver aberrations, as well as co-segregation of key recurrent alterations. For example, we observed enrichment of *IKZF1* deletions in the *BCR-ABL1* positive subgroup, common *ETV6* and *TBL1XR1* losses in patients harboring *ETV6-RUNX1* fusion, co-occurrence of *IKZF1* losses with deletions in the PAR1 and 9p regions or in the *BTG1* gene, and frequent emergence of *RB1* deletion among patients with iAMP21 genotype [29, 38–43]. The extended number of patients and the digital karyotyping probe subset of the D007 digitalMLPA probemix allowed us to identify specific chromosomal gains associated with superior outcome among patients with high-hyperdiploid karyotype, efficiently supporting the robust implementation of our personalized genetic classifier.

Deletion of the *IKZF1* gene encoding the transcription factor IKAROS is observed in 15–20% of children with B-ALL and it is reported to be associated with inferior clinical outcome with a range of different treatment protocols [27, 44–47]. *Stanulla* et al. also defined *IKZF1*^plus^, a very poor, measurable residual disease (MRD)-dependent prognostic subgroup consisting of patients who in addition to *IKZF1* loss, harbor *PAX5*, *CDKN2A/B* or PAR1 deletion, without concurrent *ERG* loss [29]. Distribution of *IKZF1* deletion patterns in our study resembles previously published data [44], with exon 4–7 deletion (isoform 6 - loss of DNA binding region) [48] being the most common subtype, followed by deletions affecting all exons with or without the analyzed upstream region. Various types of *IKZF1* loss confer inferior event-free survival regardless of their frequency and extension albeit at quite different levels of significance when compared with matching wild-type controls [49]. Although the number of patients with *IKZF1* loss was limited in our study, we observed a strong association of specific deletion patterns with either *IKZF1*^del^ or *IKZF1*^plus^ genotype. Importantly, almost all patients harboring exon 4–7 deletion showed *IKZF1*^plus^ genotype which can provide a plausible explanation why this lesion is typically associated with very adverse clinical outcome, even if patients with *IKZF1* loss are exclusively analyzed [49]. This observation certainly needs validation in larger patient cohorts prospectively or retrospectively. In terms of prognosis, our patients displaying *IKZF1* loss with or without fulfilling the criteria of *IKZF1*^plus^ showed significantly shorter EFS compared to patients without *IKZF1* deletion, nevertheless the difference between the two *IKZF1* altered subgroups were not significant, similar to a recent observation by Felice et al. [50]. Therefore, we integrated *IKZF1* status (*IKZF1*^normal^, *IKZF1*^del^ and *IKZF1*^plus^) as revealed by digitalMLPA with cytogenetic classes, thus creating a cytogenetics aware interpretation of *IKZF1* allelic status which substantially improved the risk assessment for our patients by distinguishing three prognostic groups with significantly different 5-year EFS.

Pediatric ALL develops via multi-step acquisition of molecular and cytogenetic aberrations, and a subset of alterations shows enriched co-segregation or mutual exclusivity as it has also been demonstrated in our study (Fig. 1). Nevertheless, the specific composition of detectable driver aberrations vary substantially between individual patients which is only considered to a limited extent even by the most recent prognostic classifiers [16, 26, 29, 31]. Therefore, we explored the feasibility of an alternative risk assessment approach which adaptively, at individual patient level takes into account all aberrations screened and found to be prognostically relevant in our patient cohort. This integrative method that has also been validated on a large independent patient cohort, provides important guidance not only in cases where favorable and adverse prognostic markers are simultaneously present, represented by 25% of our patients, but also allows for a more weighted prognostic classification of each individual patient and hence a refined risk assessment in general. Of note, the conventional classification applied in the ALL IC-BFM protocols failed to define three risk groups with significantly different 5-year EFS rates in our patient cohort (Fig. S7), while four distinct subgroups could be determined using PersonALL, clearly demonstrating the added value of our conceptually novel classifier. The relationships between risk groups defined by the ALL IC-BFM protocols and by the PersonALL method are outlined in Fig. S8. Excellent, good and high-risk groups of the PersonALL classification included patients from all three ALL IC-BFM risk groups and the ultra-poor risk group also involved patients originally classified in different ALL IC-BFM groups, evidently reflecting the more comprehensive principles applied in our new classifier.

Distinguishing four subgroups based on personal composition of genomic lesions as described above may have a direct impact on the clinical management of children with B-ALL in the future. Sixty percent of patients were classified into two prognostically favorable risk groups with a 5-year EFS above 85%, while the other 40% of patients were divided over two risk groups with inferior outcomes (5-year EFS below 70%). Considering the EFS rates of approximately 80-90% in pediatric ALL in the developed countries [51], we may draw some strategic conclusions with regard to the anti-leukemia treatment in these four prognostic subgroups. Reduced therapeutical intensity or longer intervals between consecutive treatment blocks may be considered for patients with “excellent” prognosis to avoid short- and long-term toxicities. MRD monitoring and MRD-driven therapy optimization should be a main focus at patients classified in the “good” and, particularly, in the “high-risk” groups. A high-resolution MRD follow-up with patient-specific molecular marker screening and monitoring would be warranted in the “high-risk” group, especially in those countries where the main MRD assessment method is flow cytometry. In cases with “ultra-poor” risk profile, treatment intensification or change in therapeutic approach (e.g. immunotherapy, cell therapy or molecularly targeted therapy) could potentially be initiated even if the early MRD response is promising, due to a very high risk of relapse during and after the first-line ALL treatment.

The current study has some limitations partially coming from the unavailability of MRD data at a significant number of patients which prevented the evaluation of an MRD-guided classifier in this retrospective analysis. Of note, our integrative genetic scoring guide aiding the classification of patients into distinct prognostic groups could still successfully be established even without accounting for the MRD status. Another shortcoming is the low abundance of some typically uncommon but prognostically relevant alterations (e.g. *TCF3-PBX1* and *NUP214-ABL1* fusions) which hampered the incorporation of those into the newly developed classifier. Nevertheless, we do believe our study provides a novel and innovative risk assessment method for patients with pediatric B-ALL, and the proposed approach, also validated on a second patient cohort, can be further refined using prospectively collected large patient populations which on one hand will overcome the limitations mentioned above, on the other hand will allow for additional independent validation of our patient-specific prognostic classifier.

In summary, our study demonstrates the power of comprehensive genomic characterization performed in a highly rationalized manner using digitalMLPA in combination with more conventional cytogenetic and molecular genetic methods. Based on the results, integration of *IKZF1* status with cytogenetic data can facilitate an enhanced IKAROS based prognostic classification which is especially well suited to the diagnostic workflow of laboratories where conventional MLPA is applied in combination with other routine assays. If NGS-based digitalMLPA or other advanced methods are available for the genome-wide characterization of disease-relevant driver aberrations, patient-specific interpretation of results is increasingly warranted and can be achieved by our conceptually novel PersonALL prognostic classifier, that can pave the way for an improved and more refined risk assessment, eventually supporting the highly personalized clinical stratification of children diagnosed with B-ALL.

## Supplementary information

Supplementary material

## Data Availability

The materials used and analyzed during the current study are available from the corresponding author on reasonable request.
