# Supplementary material for: PersonALL: a genetic scoring guide for personalized risk assessment in pediatric B-cell precursor acute lymphoblastic leukemia
Source: Br J Cancer. 2023 Jun 21;129(3):455–65. doi: 10.1038/s41416-023-02309-8 (PMC10403542; doi:10.1038/s41416-023-02309-8)
Supplement: Supplementary file 1 — Supplementary material [file 41416_2023_2309_MOESM1_ESM.pdf]

Figure S1.

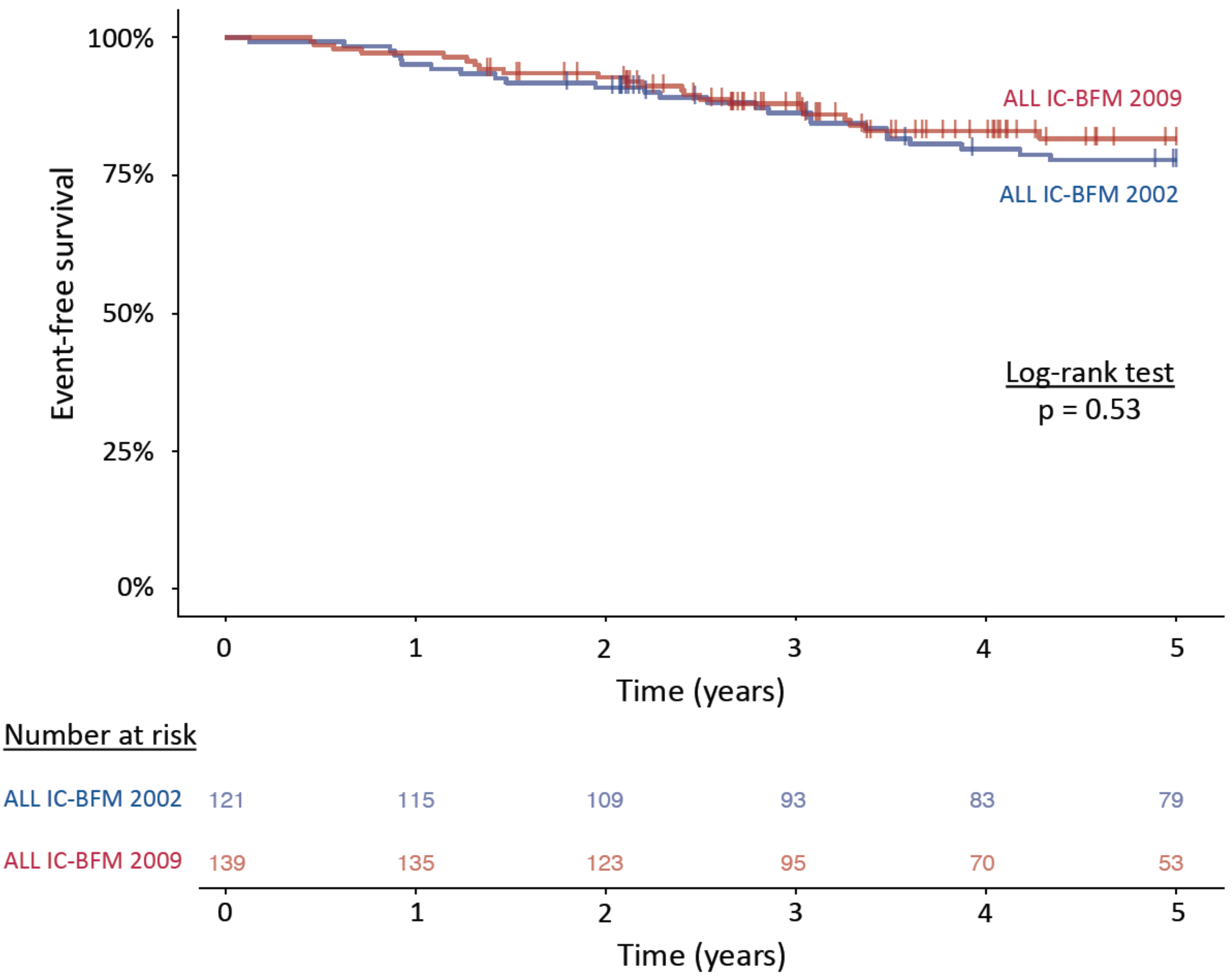

Figure S1. Event-free survival of patients treated with ALL IC-BFM 2002 and ALL IC-BFM 2009 treatment protocols. Estimated 5-year EFS did not show significant difference between the two patient cohorts (77.8% vs. 81.6%, log-rank test: p = 0.530).

Figure S2.

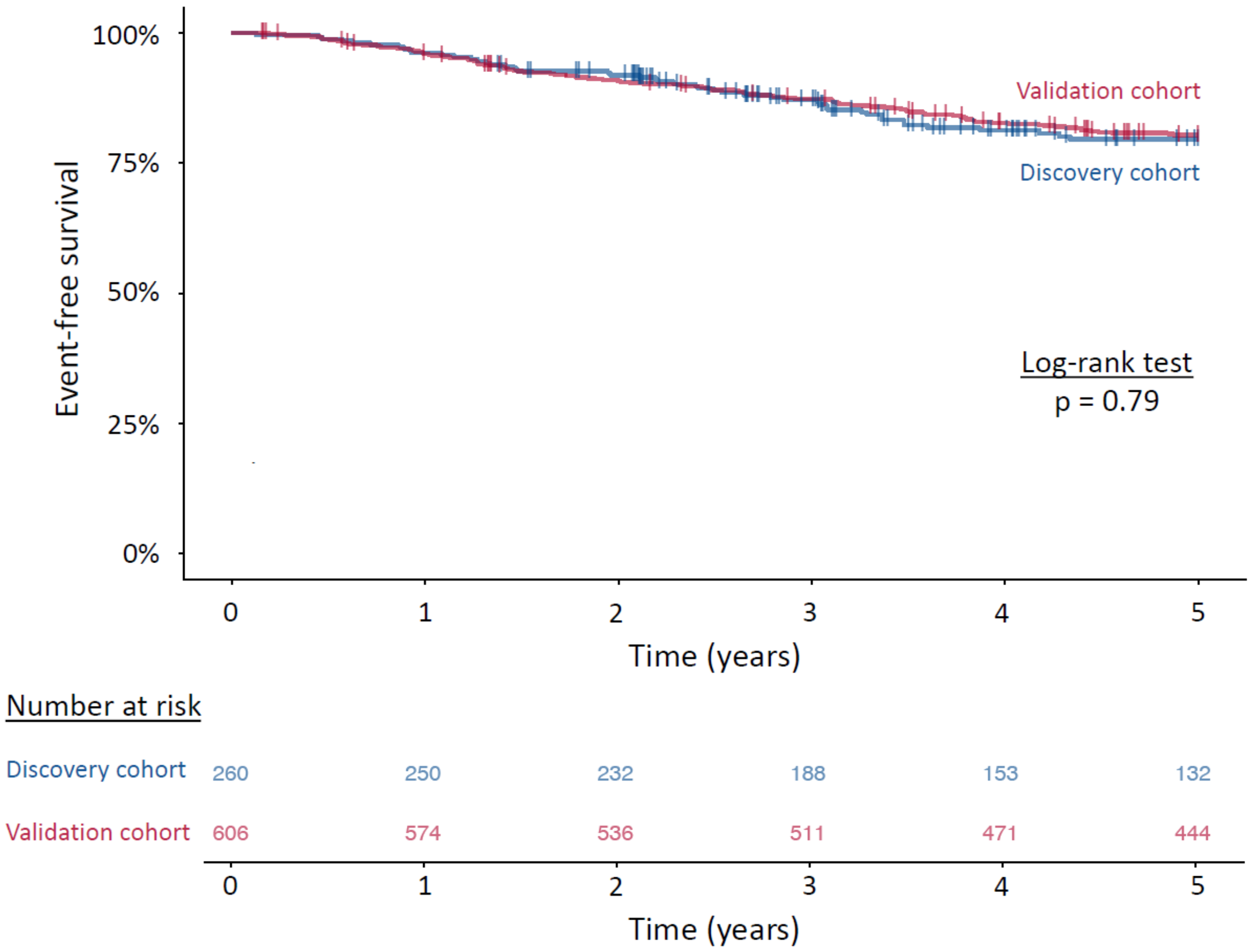

Figure S2. Event-free survival of patients included in our discovery cohort (N = 260) and in the merged TARGET ALL Phase 1 Pilot and Phase 2 Expansion cohort (N = 606). Estimated 5-year EFS did not show significant difference between the two patient cohorts (79.6% vs. 80.4%, log-rank test: p = 790).

Figure S3.

A

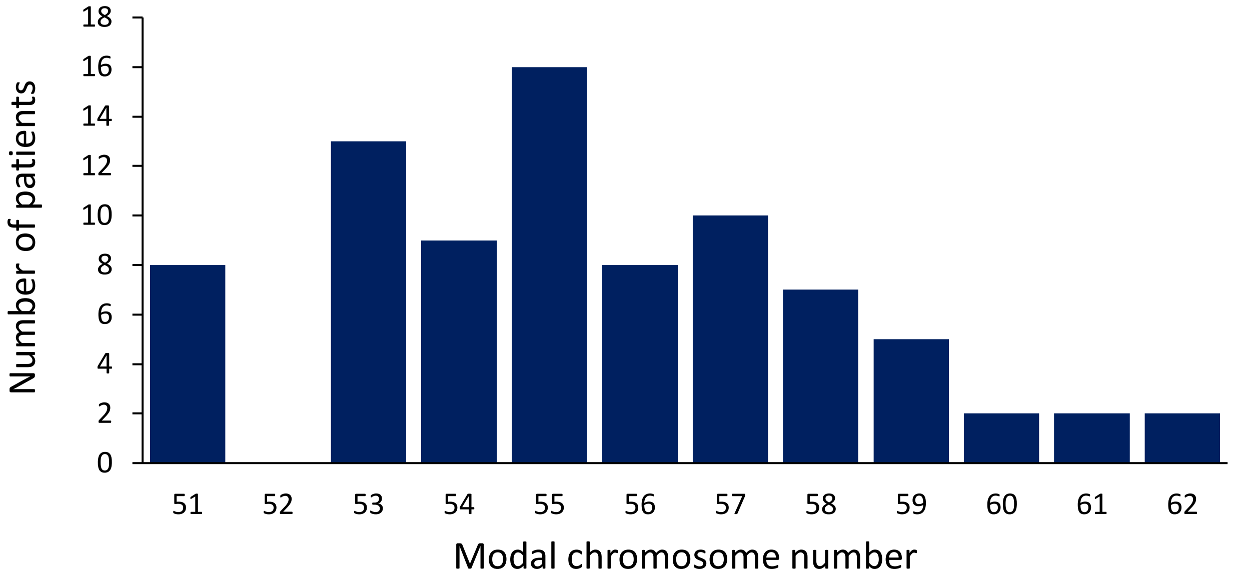

B

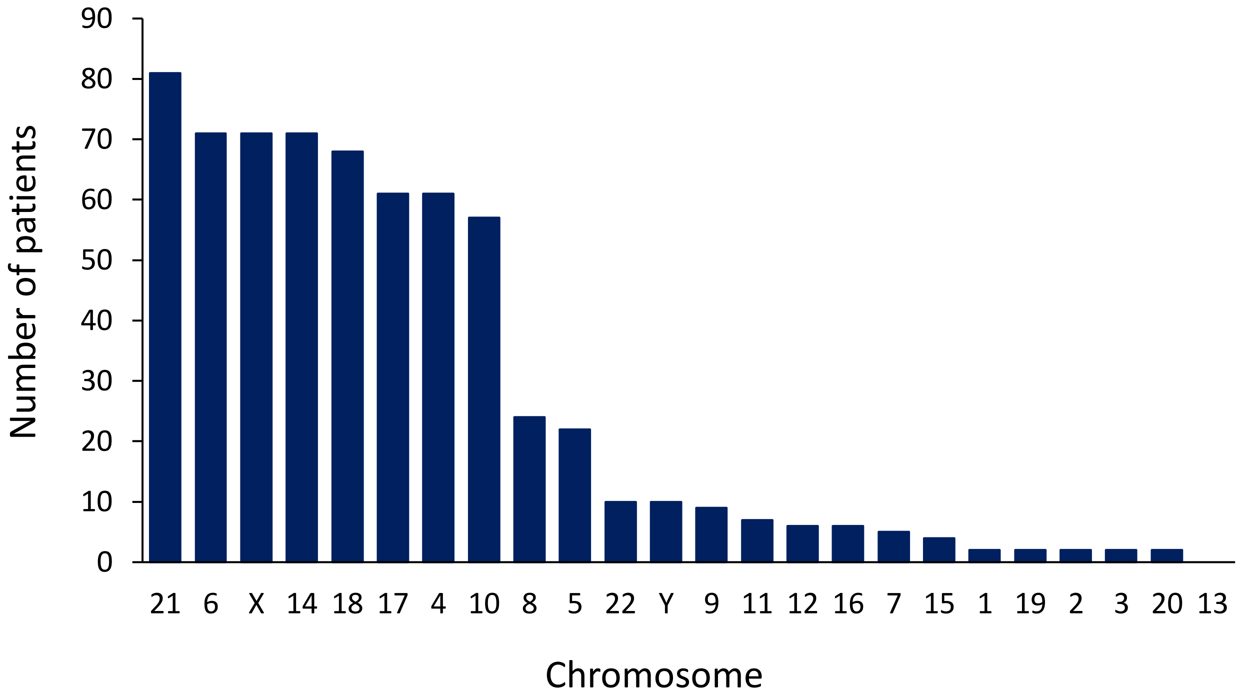

Figure S3. Modal chromosome number and frequency of whole chromosome gains in our patients with high-hyperdiploid B-cell precursor acute lymphoblastic leukemia. (A) Based on modal chromosome number determined by digitalMLPA, 82 patients displayed high-hyperdiploid karyotype with a chromosome number between 51 and 62. Multiple extra copies of chromosomes were counted as two-fold gains. (B) The pattern of chromosomal gains was dominated by extra copies of chromosomes X, 4, 6, 10, 14, 17, 18, and 21. In the diagnostic workflow, patients with hyperdiploid chromosome set were identified by karyotyping and/or DNA index measurement.

Figure S4.

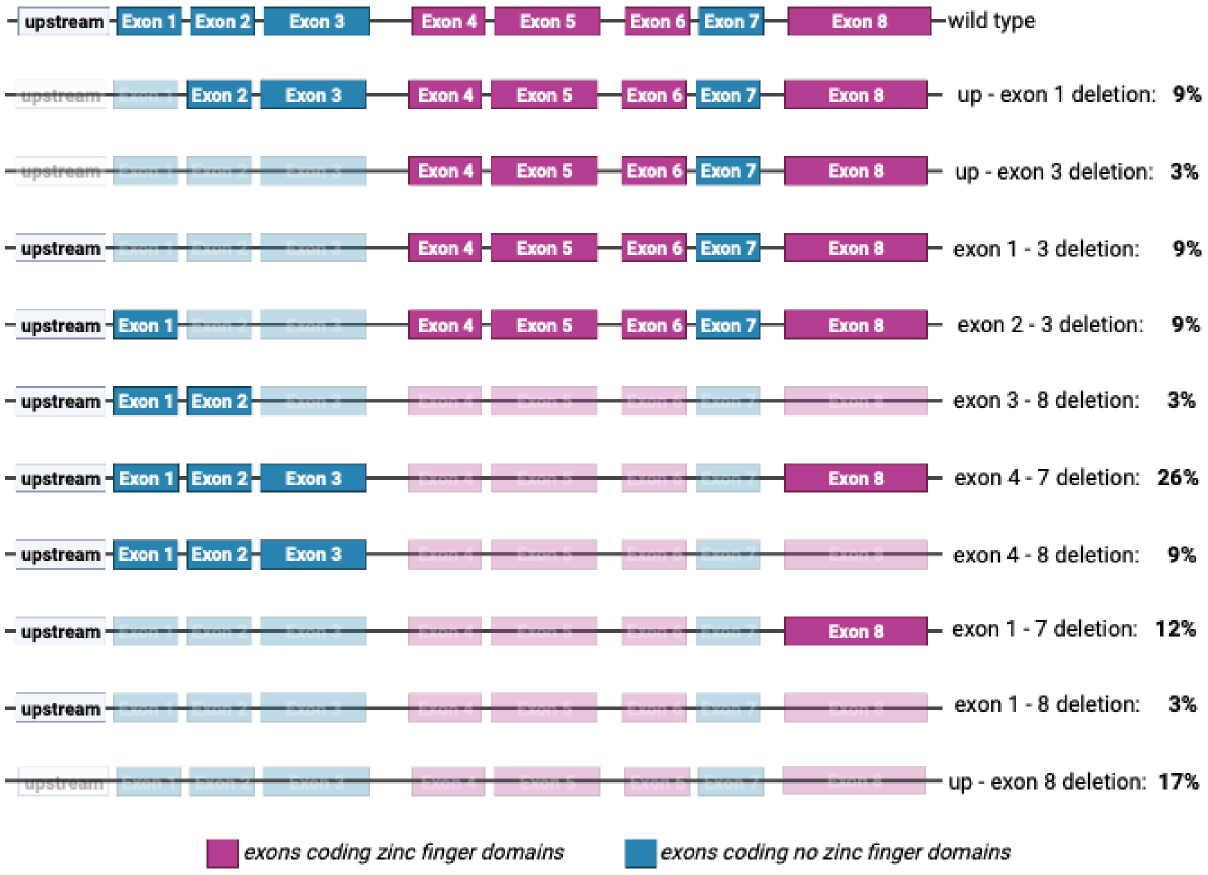

Figure S4. Patterns and frequencies of *IKZF1* gene deletions in children with B-ALL. Ten different deletion patterns were observed in 34 patients, affected exons/regions are indicated with blurred boxes. Exon 4-7 loss and upstream region/exon 1-8 deletion showed strong association with *IKZF1*^{plus} CNA profile, while exon 1-7 deletion was almost exclusively detected among patients harboring *IKZF1* loss but without meeting the criteria of *IKZF1*^{plus}.

Figure S5.

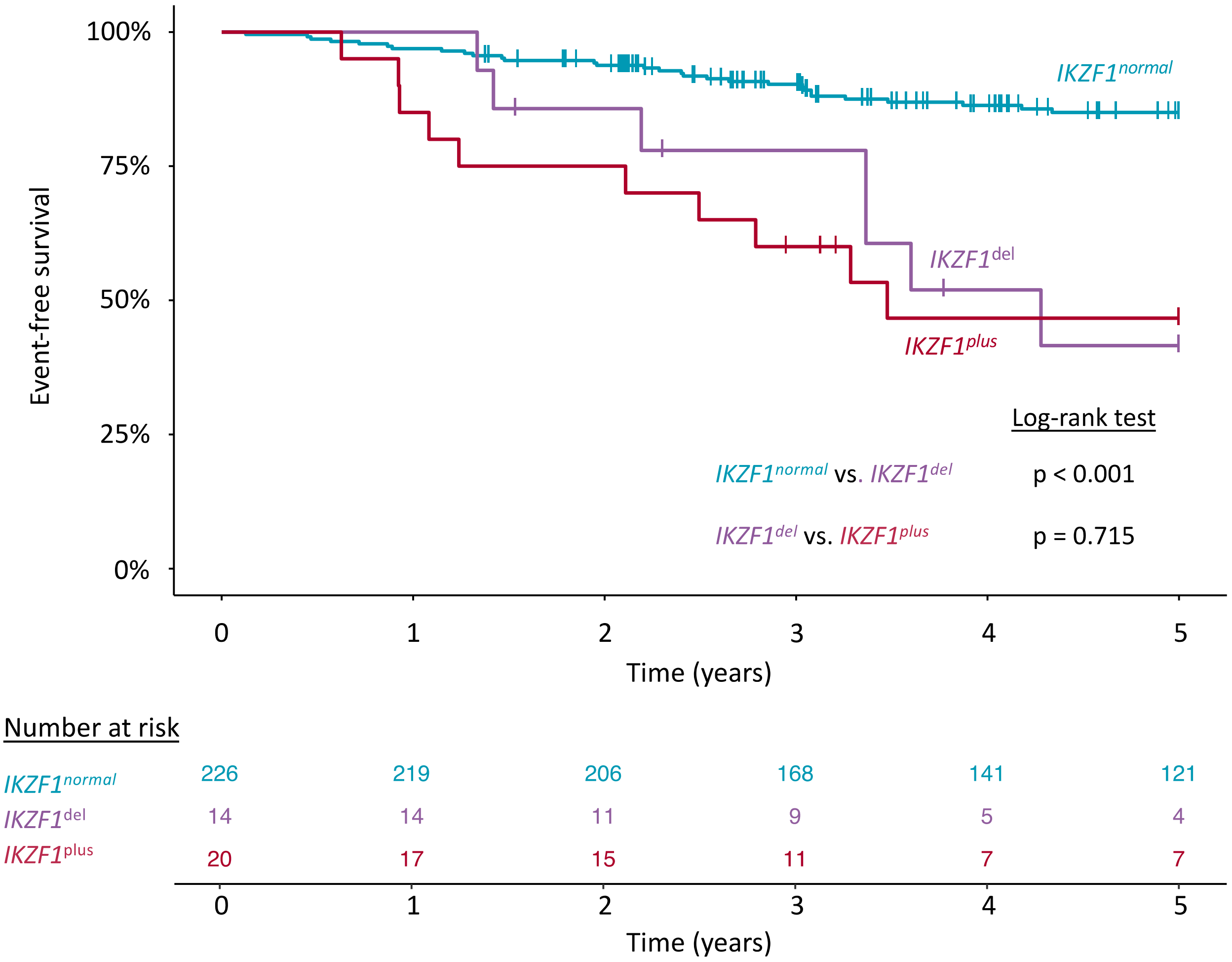

Figure S5. Event-free survival of patients with *IKZF1*^{normal}, *IKZF1*^{del} and *IKZF1*^{plus} genotypes. Patients displaying *IKZF1* deletion with or without meeting the criteria of *IKZF1*^{plus} showed shorter 5-year event-free survival as compared with patients with intact *IKZF1* alleles (*IKZF1*^{normal}: 85.0% *IKZF1*^{del}: 41.6% and *IKZF1*^{plus}: 46.7%). As in the original study by *Stanulla et al 2018,*[29] presence of *IKZF1* deletion as well as at least one additional deletion in the *PAX5*, *CDKN2A/B* or *PAR1* genes/regions in the absence of *ERG* deletion was considered as *IKZF1*^{plus} genotype.

Figure S6.

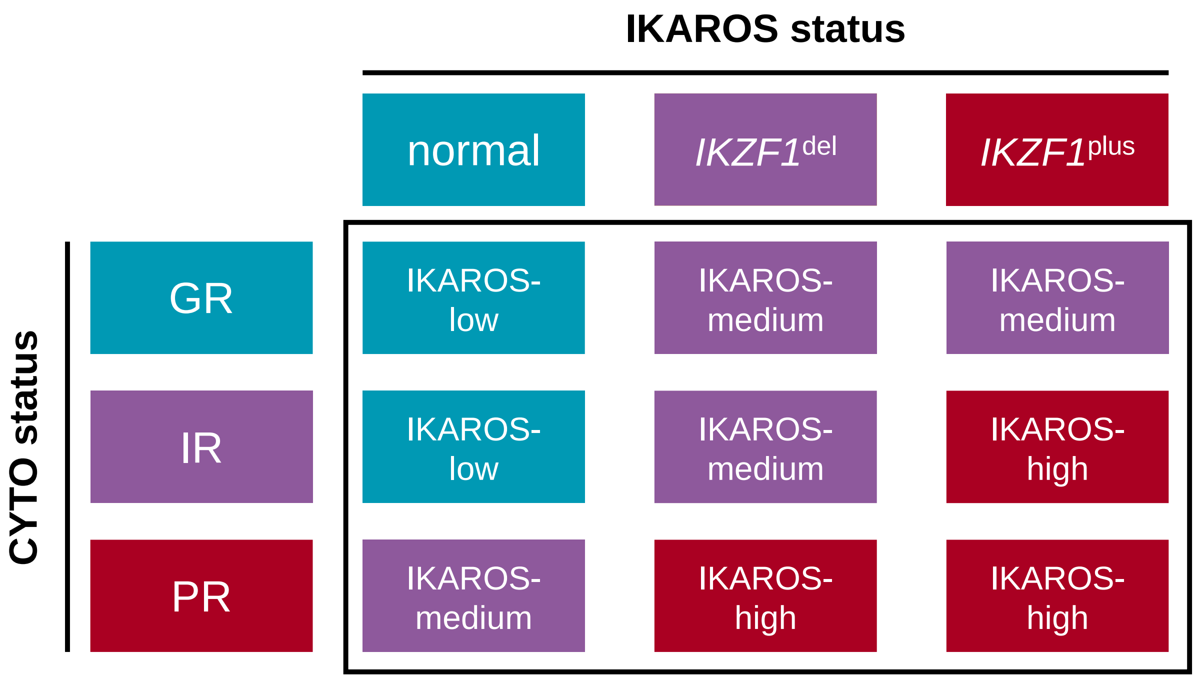

Figure S6. Combined risk groups based on cytogenetic (CYTO) features and *IKZF1* status. Cytogenetic risk groups were determined according to the UKALL system (*Hamadeh et al 2019*).[31] GR: good risk; IR: intermediate risk; PR: poor risk. *IKZF1* status is referred as *IKZF1*^{plus} if in addition to *IKZF1* loss, the patient harbored *PAX5*, *CDKN2A/B* or *PAR1* deletion, without concurrent *ERG* loss.

Figure S7.

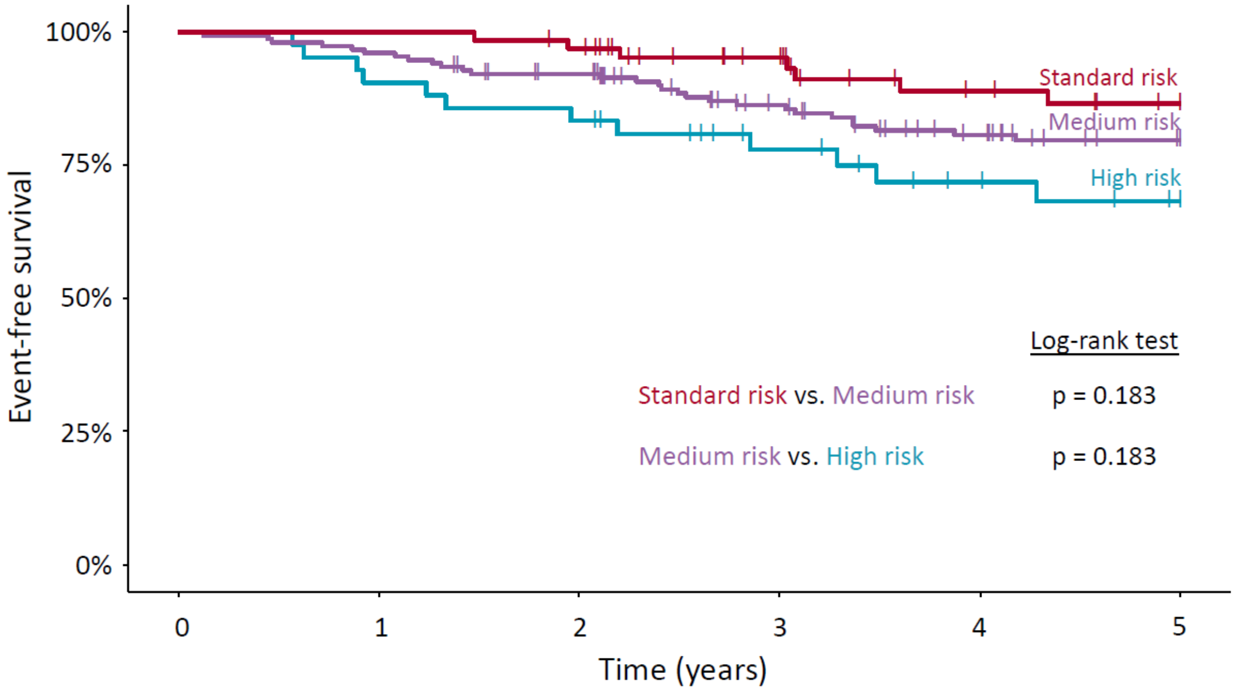

Number at risk

	0	1	2	3	4	5
Standard risk	65	65	62	50	39	34
Medium risk	153	147	135	111	93	81
High risk	42	38	35	27	21	17

Time (years)

Figure S7. Event-free survival of patients risk classified using criteria applied in the ALL IC-BFM protocols. While a clear trend was observed, difference in estimated 5-year EFS rate did not reach statistical significance between the standard and medium risk groups (86.5% vs. 79.7%, log-rank test: p = 0.183), as well as between the medium and high-risk groups (79.7% vs. 68.2%, log-rank test: p = 0.183).

Figure S8.

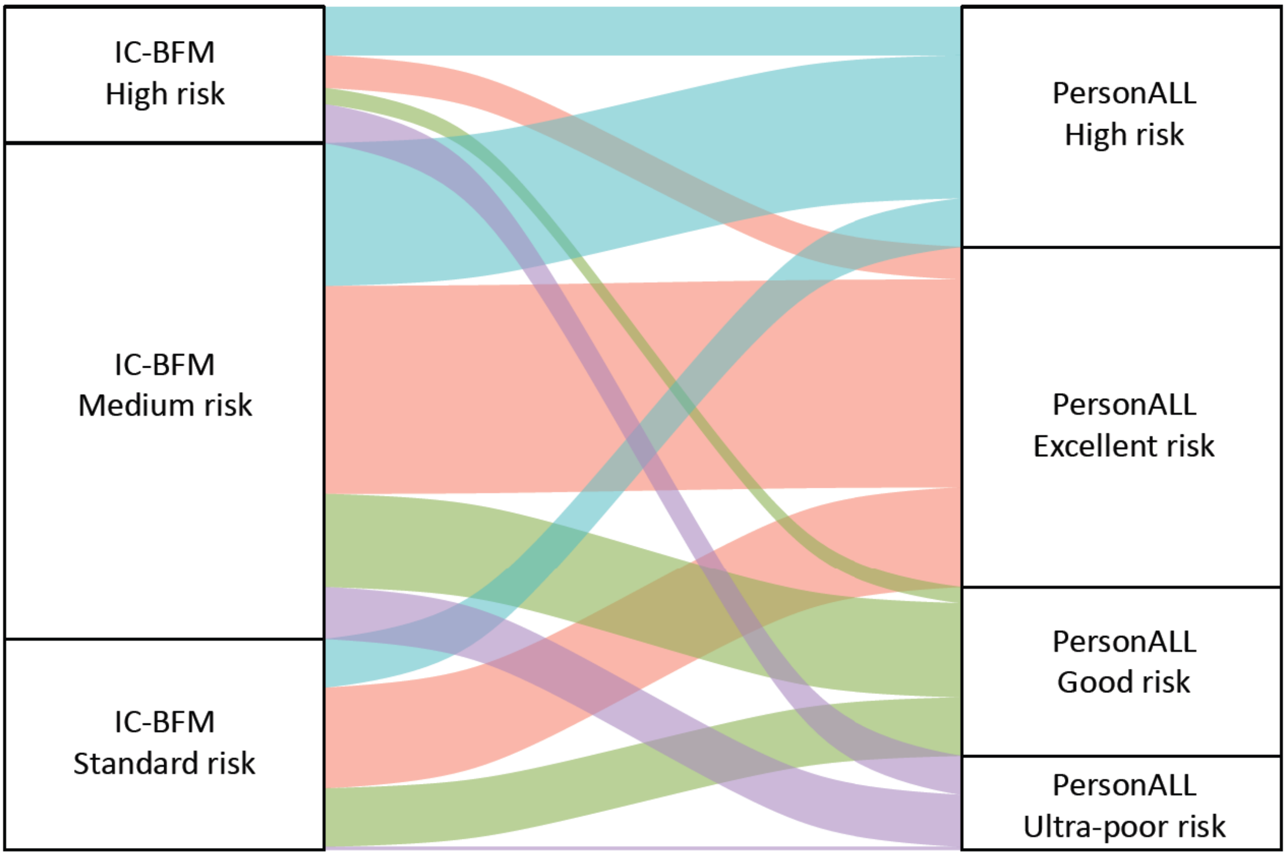

Figure S8. Assignment of patients stratified based on IC-BFM protocols to novel prognostic subgroups of PersonALL defined by non-overlapping ranges of personalized cumulative scores reflecting the weighted impact of all detected and prognostically relevant genetic aberrations. PersonALL considers all combinations of co-segregating genetic alterations, providing a more comprehensive and more refined patient stratification.

Table S1. Characteristics of patients included in our cohort.

Patient ID	Sex	Diagnosis	Age at diagnosis (years)	Blast % in bone marrow	Genetic subgroup*	Treatment protocol	Risk group***
#1	Female	B-ALL	3	94%	Hyperdiploid	ALL IC-BFM 2002	SR
#2	Male	B-ALL	11	77%	Hyperdiploid	ALL IC-BFM 2002	MR
#3	Female	B-ALL	13	79%	Hyperdiploid	ALL IC-BFM 2002	MR
#4	Male	B-ALL	5	91%	Hyperdiploid	ALL IC-BFM 2002	SR
#5	Male	B-ALL	2	35%	Hyperdiploid	ALL IC-BFM 2002	SR
#6	Male	B-ALL	15	91%	Hyperdiploid	ALL IC-BFM 2002	MR
#7	Male	B-ALL	3	93%	Hyperdiploid	ALL IC-BFM 2002	MR
#8	Female	B-ALL	4	85%	Hyperdiploid	ALL IC-BFM 2002	SR
#9	Female	B-ALL	2	81%	Hyperdiploid	ALL IC-BFM 2002	SR
#10	Male	B-ALL	4	91%	Hyperdiploid	ALL IC-BFM 2002	SR
#11	Male	B-ALL	8	68%	Hyperdiploid	ALL IC-BFM 2002	MR
#12	Female	B-ALL	3	90%	Hyperdiploid	ALL IC-BFM 2002	SR
#13	Male	B-ALL	4	80%	Hyperdiploid	ALL IC-BFM 2002	SR
#14	Male	B-ALL	3	87%	Hyperdiploid	ALL IC-BFM 2002	HR
#15	Female	B-ALL	14	97%	Hyperdiploid	ALL IC-BFM 2002	MR
#16	Female	B-ALL	6	77%	Hyperdiploid	ALL IC-BFM 2009	MR
#17	Male	B-ALL	9	85%	Hyperdiploid	ALL IC-BFM 2009	MR
#18	Male	B-ALL	3	99%	Hyperdiploid	ALL IC-BFM 2002	SR
#19	Female	B-ALL	6	91%	Hyperdiploid	ALL IC-BFM 2002	MR
#20	Male	B-ALL	4	84%	Hyperdiploid	ALL IC-BFM 2002	SR
#21	Female	B-ALL	7	95%	Hyperdiploid	ALL IC-BFM 2002	MR
#22	Male	B-ALL	2	82%	Hyperdiploid	ALL IC-BFM 2002	SR
#23	Male	B-ALL	12	87%	Hyperdiploid	ALL IC-BFM 2009	MR
#24	Female	B-ALL	14	72%	Hyperdiploid	ALL IC-BFM 2009	HR
#25	Male	B-ALL	11	86%	Hyperdiploid	ALL IC-BFM 2009	MR
#26	Male	B-ALL	2	85%	Hyperdiploid	ALL IC-BFM 2002	HR
#27	Male	B-ALL	3	78%	Hyperdiploid	ALL IC-BFM 2002	SR
#28	Female	B-ALL	2	85%	Hyperdiploid	ALL IC-BFM 2002	SR
#29	Male	B-ALL	4	82%	Hyperdiploid	ALL IC-BFM 2009	MR
#30	Male	B-ALL	10	92%	Hyperdiploid	ALL IC-BFM 2002	MR
#31	Male	B-ALL	2	85%	Hyperdiploid	ALL IC-BFM 2009	HR
#32	Male	B-ALL	5	92%	Hyperdiploid	ALL IC-BFM 2009	MR
#33	Male	B-ALL	3	83%	Hyperdiploid	ALL IC-BFM 2009	MR
#34	Female	B-ALL	2	95%	Hyperdiploid	ALL IC-BFM 2002	HR
#35	Female	B-ALL	3	68%	Hyperdiploid	ALL IC-BFM 2009	MR
#36	Male	B-ALL	3	83%	Hyperdiploid	ALL IC-BFM 2009	HR
#37	Male	B-ALL	3	81%	Hyperdiploid	ALL IC-BFM 2009	HR
#38	Male	B-ALL	5	83%	Hyperdiploid	ALL IC-BFM 2009	MR
#39	Female	B-ALL	5	90%	Hyperdiploid	ALL IC-BFM 2002	MR
#40	Female	B-ALL	7	74%	Hyperdiploid	ALL IC-BFM 2009	MR
#41	Male	B-ALL	3	51%	Hyperdiploid	ALL IC-BFM 2009	SR
#42	Female	B-ALL	15	55%	Hyperdiploid	ALL IC-BFM 2009	MR
#43	Male	B-ALL	7	86%	Hyperdiploid	ALL IC-BFM 2009	MR

#44	Female	B-ALL	4	95%	Hyperdiploid	ALL IC-BFM 2009	SR
#45	Male	B-ALL	6	77%	Hyperdiploid	ALL IC-BFM 2009	MR
#46	Female	B-ALL	3	78%	Hyperdiploid	ALL IC-BFM 2009	MR
#47	Female	B-ALL	5	88%	Hyperdiploid	ALL IC-BFM 2002	SR
#48	Male	B-ALL	4	85%	Hyperdiploid	ALL IC-BFM 2002	MR
#49	Male	B-ALL	3	82%	Hyperdiploid	ALL IC-BFM 2002	SR
#50	Male	B-ALL	6	93%	Hyperdiploid	ALL IC-BFM 2002	MR
#51	Female	B-ALL	2	90%	Hyperdiploid	ALL IC-BFM 2002	HR
#52	Female	B-ALL	3	95%	Hyperdiploid	ALL IC-BFM 2009	MR
#53	Male	B-ALL	9	91%	Hyperdiploid	ALL IC-BFM 2009	MR
#54	Male	B-ALL	3	87%	Hyperdiploid	ALL IC-BFM 2002	MR
#55	Female	B-ALL	5	90%	Hyperdiploid	ALL IC-BFM 2002	SR
#56	Female	B-ALL	5	85%	Hyperdiploid	ALL IC-BFM 2009	SR
#57	Male	B-ALL	9	69%	Hyperdiploid	ALL IC-BFM 2002	MR
#58	Male	B-ALL	4	82%	Hyperdiploid	ALL IC-BFM 2002	SR
#59	Female	B-ALL	16	95%	Hyperdiploid	ALL IC-BFM 2009	HR
#60	Female	B-ALL	3	72%	Hyperdiploid	ALL IC-BFM 2009	MR
#61	Male	B-ALL	5	94%	Hyperdiploid	ALL IC-BFM 2009	MR
#62	Female	B-ALL	5	96%	Hyperdiploid	ALL IC-BFM 2009	MR
#63	Male	B-ALL	9	56%	Hyperdiploid	ALL IC-BFM 2009	MR
#64	Female	B-ALL	2	69%	Hyperdiploid	ALL IC-BFM 2009	MR
#65	Female	B-ALL	6	76%	Hyperdiploid	ALL IC-BFM 2009	SR
#66	Female	B-ALL	4	94%	Hyperdiploid	ALL IC-BFM 2009	MR
#67	Female	B-ALL	2	74%	Hyperdiploid	ALL IC-BFM 2009	MR
#68	Male	B-ALL	2	90%	Hyperdiploid	ALL IC-BFM 2009	MR
#69	Male	B-ALL	4	63%	Hyperdiploid	ALL IC-BFM 2009	HR
#70	Female	B-ALL	4	67%	Hyperdiploid	ALL IC-BFM 2009	HR
#71	Male	B-ALL	17	51%	Hyperdiploid	ALL IC-BFM 2009	MR
#72	Female	B-ALL	4	92%	Hyperdiploid	ALL IC-BFM 2009	MR
#73	Female	B-ALL	8	84%	Hyperdiploid	ALL IC-BFM 2009	MR
#74	Male	B-ALL	3	61%	Hyperdiploid	ALL IC-BFM 2002	MR
#75	Female	B-ALL	4	71%	Hyperdiploid	ALL IC-BFM 2009	SR
#76	Female	B-ALL	3	92%	Hyperdiploid	ALL IC-BFM 2009	MR
#77	Female	B-ALL	8	89%	Hyperdiploid	ALL IC-BFM 2009	MR
#78	Male	B-ALL	16	84%	Hyperdiploid	ALL IC-BFM 2009	MR
#79	Male	B-ALL	4	90%	Hyperdiploid	ALL IC-BFM 2009	MR
#80	Male	B-ALL	3	92%	Hyperdiploid	ALL IC-BFM 2009	MR
#81	Male	B-ALL	1	96%	Hyperdiploid	ALL IC-BFM 2009	MR
#82	Female	B-ALL	4	85%	Hyperdiploid and *ETV6-RUNX1*	ALL IC-BFM 2009	MR
#83	Male	B-ALL	16	50%	Hyperdiploid and *BCR-ABL1*	ALL IC-BFM 2009	HR
#84	Female	B-ALL	10	87%	*ETV6-RUNX1*	ALL IC-BFM 2002	MR
#85	Male	B-ALL	2	82%	*ETV6-RUNX1*	ALL IC-BFM 2002	SR
#86	Male	B-ALL	9	92%	*ETV6-RUNX1*	ALL IC-BFM 2002	MR
#87	Female	B-ALL	7	93%	*ETV6-RUNX1*	ALL IC-BFM 2002	MR

#88	Male	B-ALL	6	84%	*ETV6-RUNX1*	ALL IC-BFM 2002	MR
#89	Male	B-ALL	4	82%	*ETV6-RUNX1*	ALL IC-BFM 2002	MR
#90	Male	B-ALL	4	75%	*ETV6-RUNX1*	ALL IC-BFM 2002	MR
#91	Male	B-ALL	2	73%	*ETV6-RUNX1*	ALL IC-BFM 2002	SR
#92	Male	B-ALL	5	88%	*ETV6-RUNX1*	ALL IC-BFM 2002	MR
#93	Female	B-ALL	2	92%	*ETV6-RUNX1*	ALL IC-BFM 2002	MR
#94	Male	B-ALL	6	90%	*ETV6-RUNX1*	ALL IC-BFM 2002	SR
#95	Male	B-ALL	3	93%	*ETV6-RUNX1*	ALL IC-BFM 2002	MR
#96	Female	B-ALL	6	94%	*ETV6-RUNX1*	ALL IC-BFM 2002	MR
#97	Female	B-ALL	2	60%	*ETV6-RUNX1*	ALL IC-BFM 2002	MR
#98	Female	B-ALL	3	68%	*ETV6-RUNX1*	ALL IC-BFM 2002	SR
#99	Male	B-ALL	4	91%	*ETV6-RUNX1*	ALL IC-BFM 2002	SR
#100	Female	B-ALL	2	79%	*ETV6-RUNX1*	ALL IC-BFM 2002	MR
#101	Male	B-ALL	6	62%	*ETV6-RUNX1*	ALL IC-BFM 2002	HR
#102	Female	B-ALL	4	88%	*ETV6-RUNX1*	ALL IC-BFM 2002	SR
#103	Male	B-ALL	6	73%	*ETV6-RUNX1*	ALL IC-BFM 2002	MR
#104	Male	B-ALL	1	79%	*ETV6-RUNX1*	ALL IC-BFM 2002	MR
#105	Male	B-ALL	9	84%	*ETV6-RUNX1*	ALL IC-BFM 2009	MR
#106	Female	B-ALL	5	83%	*ETV6-RUNX1*	ALL IC-BFM 2009	MR
#107	Male	B-ALL	3	80%	*ETV6-RUNX1*	ALL IC-BFM 2002	SR
#108	Male	B-ALL	5	33%	*ETV6-RUNX1*	ALL IC-BFM 2009	SR
#109	Male	B-ALL	5	65%	*ETV6-RUNX1*	ALL IC-BFM 2002	SR
#110	Female	B-ALL	5	73%	*ETV6-RUNX1*	ALL IC-BFM 2002	MR
#111	Female	B-ALL	2	49%	*ETV6-RUNX1*	ALL IC-BFM 2009	MR
#112	Male	B-ALL	4	61%	*ETV6-RUNX1*	ALL IC-BFM 2009	MR
#113	Female	B-ALL	3	57%	*ETV6-RUNX1*	ALL IC-BFM 2009	SR
#114	Female	B-ALL	16	94%	*ETV6-RUNX1*	ALL IC-BFM 2009	MR
#115	Male	B-ALL	7	97%	*ETV6-RUNX1*	ALL IC-BFM 2009	MR
#116	Male	B-ALL	4	60%	*ETV6-RUNX1*	ALL IC-BFM 2009	MR
#117	Male	B-ALL	6	83%	*ETV6-RUNX1*	ALL IC-BFM 2009	MR
#118	Male	B-ALL	5	77%	*ETV6-RUNX1*	ALL IC-BFM 2009	MR
#119	Female	B-ALL	5	94%	*ETV6-RUNX1*	ALL IC-BFM 2002	MR
#120	Male	B-ALL	4	86%	*ETV6-RUNX1*	ALL IC-BFM 2002	MR
#121	Female	B-ALL	9	81%	*ETV6-RUNX1*	ALL IC-BFM 2002	MR
#122	Male	B-ALL	4	96%	*ETV6-RUNX1*	ALL IC-BFM 2002	SR
#123	Female	B-ALL	9	88%	*ETV6-RUNX1*	ALL IC-BFM 2009	MR
#124	Female	B-ALL	6	46%	*ETV6-RUNX1*	ALL IC-BFM 2009	HR
#125	Male	B-ALL	4	83%	*ETV6-RUNX1*	ALL IC-BFM 2009	SR
#126	Male	B-ALL	4	91%	*ETV6-RUNX1*	ALL IC-BFM 2009	SR
#127	Male	B-ALL	3	71%	*ETV6-RUNX1*	ALL IC-BFM 2009	SR
#128	Male	B-ALL	11	80%	*ETV6-RUNX1*	ALL IC-BFM 2002	MR
#129	Female	B-ALL	5	90%	*ETV6-RUNX1*	ALL IC-BFM 2002	SR
#130	Male	B-ALL	5	92%	*ETV6-RUNX1*	ALL IC-BFM 2009	SR
#131	Male	B-ALL	5	52%	*ETV6-RUNX1*	ALL IC-BFM 2002	SR
#132	Male	B-ALL	2	88%	*ETV6-RUNX1*	ALL IC-BFM 2009	MR
#133	Male	B-ALL	5	88%	*ETV6-RUNX1*	ALL IC-BFM 2009	MR
#134	Female	B-ALL	11	89%	*ETV6-RUNX1*	ALL IC-BFM 2009	HR
#135	Male	B-ALL	4	58%	*ETV6-RUNX1*	ALL IC-BFM 2009	MR

#136	Female	B-ALL	4	89%	*ETV6-RUNX1*	ALL IC-BFM 2002	MR
#137	Female	B-ALL	2	63%	*ETV6-RUNX1*	ALL IC-BFM 2009	MR
#138	Male	B-ALL	12	93%	*ETV6-RUNX1*	ALL IC-BFM 2002	MR
#139	Female	B-ALL	13	90%	*ETV6-RUNX1*	ALL IC-BFM 2009	MR
#140	Female	B-ALL	4	54%	*ETV6-RUNX1*	ALL IC-BFM 2009	SR
#141	Female	B-ALL	4	93%	*ETV6-RUNX1*	ALL IC-BFM 2009	SR
#142	Male	B-ALL	6	95%	*ETV6-RUNX1*	ALL IC-BFM 2009	MR
#143	Male	B-ALL	10	96%	*ETV6-RUNX1*	ALL IC-BFM 2009	MR
#144	Female	B-ALL	2	92%	*ETV6-RUNX1*	ALL IC-BFM 2009	MR
#145	Male	B-ALL	6	95%	*ETV6-RUNX1*	ALL IC-BFM 2009	MR
#146	Male	B-ALL	3	95%	*ETV6-RUNX1*	ALL IC-BFM 2009	MR
#147	Female	B-ALL	3	60%	*ETV6-RUNX1*	ALL IC-BFM 2009	MR
#148	Male	B-ALL	3	61%	*ETV6-RUNX1*	ALL IC-BFM 2009	SR
#149	Female	B-ALL	5	49%	*ETV6-RUNX1*	ALL IC-BFM 2009	MR
#150	Female	B-ALL	2	29%	*ETV6-RUNX1*	ALL IC-BFM 2009	MR
#151	Male	B-ALL	16	52%	*BCR-ABL1*	ALL IC-BFM 2002	HR
#152	Male	B-ALL	2	89%	*BCR-ABL1*	ALL IC-BFM 2002	HR
#153	Male	B-ALL	2	75%	*BCR-ABL1*	ALL IC-BFM 2002	HR
#154	Male	B-ALL	6	81%	*BCR-ABL1*	ALL IC-BFM 2002	HR
#155	Male	B-ALL	9	94%	*BCR-ABL1* and iAMP21	ALL IC-BFM 2009	HR
#156	Male	B-ALL	12	94%	iAMP21	ALL IC-BFM 2002	MR
#157	Male	B-ALL	9	74%	iAMP21	ALL IC-BFM 2002	MR
#158	Female	B-ALL	11	58%	iAMP21	ALL IC-BFM 2002	MR
#159	Male	B-ALL	15	82%	iAMP21	ALL IC-BFM 2002	MR
#160	Male	B-ALL	7	43%	iAMP21	ALL IC-BFM 2002	HR
#161	Male	B-ALL	12	90%	iAMP21	ALL IC-BFM 2009	MR
#162	Male	B-ALL	9	40%	iAMP21	ALL IC-BFM 2009	MR
#163	Male	B-ALL	8	82%	iAMP21	ALL IC-BFM 2009	HR
#164	Female	B-ALL	9	93%	iAMP21	ALL IC-BFM 2009	MR
#165	Female	B-ALL	15	67%	*KMT2A*r	ALL IC-BFM 2002	MR
#166	Female	B-ALL	5	86%	*KMT2A*r	ALL IC-BFM 2002	MR
#167	Female	B-ALL	2	88%	*KMT2A*r	ALL IC-BFM 2009	HR
#168	Female	B-ALL	1	40%	*KMT2A*r	ALL IC-BFM 2009	MR
#169	Female	B-ALL	1	94%	*TCF3-PBX1*	ALL IC-BFM 2002	MR
#170	Male	B-ALL	8	64%	*TCF3-PBX1*	ALL IC-BFM 2009	MR
#171	Male	B-ALL	4	85%	*TCF3-PBX1*	ALL IC-BFM 2009	HR
#172	Male	B-ALL	16	87%	B-other**	ALL IC-BFM 2002	MR
#173	Female	B-ALL	2	61%	B-other	ALL IC-BFM 2002	MR
#174	Female	B-ALL	3	70%	B-other	ALL IC-BFM 2002	MR
#175	Male	B-ALL	4	87%	B-other	ALL IC-BFM 2002	MR
#176	Female	B-ALL	11	84%	B-other	ALL IC-BFM 2002	HR
#177	Male	B-ALL	5	84%	B-other	ALL IC-BFM 2002	SR
#178	Male	B-ALL	5	78%	B-other	ALL IC-BFM 2002	SR
#179	Female	B-ALL	4	97%	B-other	ALL IC-BFM 2002	MR
#180	Male	B-ALL	10	75%	B-other	ALL IC-BFM 2002	MR
#181	Female	B-ALL	11	59%	B-other	ALL IC-BFM 2002	HR
#182	Female	B-ALL	9	78%	B-other	ALL IC-BFM 2002	MR

#183	Female	B-ALL	3	70%	B-other	ALL IC-BFM 2002	SR
#184	Female	B-ALL	4	73%	B-other	ALL IC-BFM 2002	SR
#185	Female	B-ALL	3	97%	B-other	ALL IC-BFM 2002	SR
#186	Male	B-ALL	14	84%	B-other	ALL IC-BFM 2009	MR
#187	Male	B-ALL	4	94%	B-other	ALL IC-BFM 2002	MR
#188	Female	B-ALL	13	72%	B-other	ALL IC-BFM 2002	MR
#189	Male	B-ALL	5	66%	B-other	ALL IC-BFM 2002	HR
#190	Female	B-ALL	13	92%	B-other	ALL IC-BFM 2009	HR
#191	Male	B-ALL	13	87%	B-other	ALL IC-BFM 2009	MR
#192	Male	B-ALL	17	93%	B-other	ALL IC-BFM 2009	MR
#193	Male	B-ALL	4	80%	B-other	ALL IC-BFM 2002	SR
#194	Male	B-ALL	4	57%	B-other	ALL IC-BFM 2009	SR
#195	Male	B-ALL	7	96%	B-other	ALL IC-BFM 2009	MR
#196	Male	B-ALL	3	58%	B-other	ALL IC-BFM 2009	SR
#197	Male	B-ALL	10	30%	B-other	ALL IC-BFM 2009	MR
#198	Female	B-ALL	1	43%	B-other	ALL IC-BFM 2009	MR
#199	Female	B-ALL	6	77%	B-other	ALL IC-BFM 2009	HR
#200	Male	B-ALL	13	89%	B-other	ALL IC-BFM 2009	HR
#201	Female	B-ALL	10	59%	B-other	ALL IC-BFM 2002	MR
#202	Female	B-ALL	1	74%	B-other	ALL IC-BFM 2002	SR
#203	Male	B-ALL	3	86%	B-other	ALL IC-BFM 2002	MR
#204	Male	B-ALL	6	58%	B-other	ALL IC-BFM 2002	SR
#205	Male	B-ALL	11	75%	B-other	ALL IC-BFM 2002	MR
#206	Female	B-ALL	2	86%	B-other	ALL IC-BFM 2002	SR
#207	Female	B-ALL	4	88%	B-other	ALL IC-BFM 2002	SR
#208	Female	B-ALL	4	69%	B-other	ALL IC-BFM 2009	HR
#209	Female	B-ALL	3	46%	B-other	ALL IC-BFM 2009	SR
#210	Male	B-ALL	12	90%	B-other	ALL IC-BFM 2009	MR
#211	Male	B-ALL	9	50%	B-other	ALL IC-BFM 2009	MR
#212	Female	B-ALL	3	58%	B-other	ALL IC-BFM 2009	SR
#213	Male	B-ALL	4	68%	B-other	ALL IC-BFM 2009	SR
#214	Female	B-ALL	12	88%	B-other	ALL IC-BFM 2002	MR
#215	Male	B-ALL	2	89%	B-other	ALL IC-BFM 2009	MR
#216	Male	B-ALL	12	92%	B-other	ALL IC-BFM 2002	MR
#217	Female	B-ALL	5	95%	B-other	ALL IC-BFM 2009	HR
#218	Male	B-ALL	3	47%	B-other	ALL IC-BFM 2009	MR
#219	Male	B-ALL	6	91%	B-other	ALL IC-BFM 2009	MR
#220	Male	B-ALL	2	87%	B-other	ALL IC-BFM 2009	MR
#221	Female	B-ALL	15	60%	B-other	ALL IC-BFM 2009	HR
#222	Female	B-ALL	9	87%	B-other	ALL IC-BFM 2009	HR
#223	Male	B-ALL	9	72%	B-other	ALL IC-BFM 2002	HR
#224	Male	B-ALL	2	60%	B-other	ALL IC-BFM 2002	SR
#225	Female	B-ALL	10	83%	B-other	ALL IC-BFM 2002	MR
#226	Male	B-ALL	17	82%	B-other	ALL IC-BFM 2002	MR
#227	Male	B-ALL	8	82%	B-other	ALL IC-BFM 2009	MR
#228	Male	B-ALL	3	85%	B-other	ALL IC-BFM 2009	HR
#229	Female	B-ALL	2	80%	B-other	ALL IC-BFM 2009	MR
#230	Male	B-ALL	13	82%	B-other	ALL IC-BFM 2002	HR

#231	Male	B-ALL	4	85%	B-other	ALL IC-BFM 2002	SR
#232	Male	B-ALL	1	85%	B-other	ALL IC-BFM 2002	MR
#233	Male	B-ALL	8	94%	B-other	ALL IC-BFM 2002	MR
#234	Male	B-ALL	3	80%	B-other	ALL IC-BFM 2009	SR
#235	Female	B-ALL	17	85%	B-other	ALL IC-BFM 2002	MR
#236	Male	B-ALL	8	92%	B-other	ALL IC-BFM 2002	MR
#237	Male	B-ALL	16	69%	B-other	ALL IC-BFM 2009	HR
#238	Female	B-ALL	3	61%	B-other	ALL IC-BFM 2009	HR
#239	Male	B-ALL	9	90%	B-other	ALL IC-BFM 2009	HR
#240	Male	B-ALL	15	82%	B-other	ALL IC-BFM 2009	MR
#241	Male	B-ALL	12	54%	B-other	ALL IC-BFM 2009	HR
#242	Male	B-ALL	16	88%	B-other	ALL IC-BFM 2009	MR
#243	Male	B-ALL	10	80%	B-other	ALL IC-BFM 2009	MR
#244	Male	B-ALL	3	89%	B-other	ALL IC-BFM 2009	MR
#245	Female	B-ALL	2	84%	B-other	ALL IC-BFM 2009	MR
#246	Male	B-ALL	5	91%	B-other	ALL IC-BFM 2009	MR
#247	Female	B-ALL	4	81%	B-other	ALL IC-BFM 2002	SR
#248	Male	B-ALL	8	96%	B-other	ALL IC-BFM 2009	MR
#249	Female	B-ALL	6	96%	B-other	ALL IC-BFM 2009	SR
#250	Male	B-ALL	10	95%	B-other	ALL IC-BFM 2009	MR
#251	Male	B-ALL	8	79%	B-other	ALL IC-BFM 2009	MR
#252	Male	B-ALL	3	92%	B-other	ALL IC-BFM 2002	SR
#253	Male	B-ALL	4	94%	B-other	ALL IC-BFM 2002	SR
#254	Female	B-ALL	3	90%	B-other	ALL IC-BFM 2009	MR
#255	Male	B-ALL	2	40%	B-other	ALL IC-BFM 2009	MR
#256	Female	B-ALL	13	67%	B-other	ALL IC-BFM 2009	MR
#257	Male	B-ALL	17	97%	B-other	ALL IC-BFM 2009	MR
#258	Female	B-ALL	4	95%	B-other	ALL IC-BFM 2009	MR
#259	Male	B-ALL	1	62%	B-other	ALL IC-BFM 2009	HR
#260	Female	B-ALL	6	98%	B-other	ALL IC-BFM 2009	SR

* Determined by karyotyping, DNA-index measurement and fluorescence *in situ* hybridization
** Patients negative for hyperdiploidy, hypodiploidy, *ETV6-RUNX1* fusion, *BCR-ABL1* fusion, iAMP21, *KMT2Ar* and *TCF3-PBX1*
***SR: standard risk, MR: medium risk, HR: high risk

Table S2. Genes recurrently altered in acute lymphoblastic leukemia and covered by the D007-X2-0516 and D007-X5-0220 digitalMLPA probemixes.

D007-X2-0516			
**Chromosome region**	**Gene**	**Exons**	**Reference**
1p33	*TAL1*	1,3,5,6	NM_003189.5
1p33	*STIL*	1,2,6,12	NM_001048166.1
2q34	*IKZF2*	2,5,9	NM_016260.2
3q13.2	*CD200*	3,4,6	NM_005944.5
3q13.2	*BTLA*	1,2,5	NM_181780.3
3q26.32	*TBL1XR1*	upstream, 1,2,7,15	NM_024665.4
4q25	*LEF1*	1,3,4,7,12	NM_016269.4
4q31.23	*NR3C2*	2,4,9	NM_000901.4
5q31.3	*NR3C1*	Upstream,1,2,5,8	NM_001018077.1
5q32	*PDGFRB*	9,10,11,12	NM_002609.3
5q33.3	*EBF1*	8,10,14,15,16	NM_001290360.1
6q15	*CASP8AP2*	1,2,6,10	NM_012115.3
6q23.3	*MYB*	2,6,16	NM_001130173
7p12.2	*IKZF1*	Upstream,1,2,3,4,5,6,7,8 and alternative exon 1 (*NM_001291837.1)*	NM_006060.5
7q36.1	*EZH2*	2,15,20	NM_004456.4
9p21.3	*MLLT3*	2,5,7	NM_004529.3
9p21.3	*CDKN2A*	Upstream,1,2 and alternative exon 3 (*NM_000077.4)* and exon 4 (*NM_001195132*)	NM_058195.3
9p21.3	*CDKN2B*	1,2	NM_078487.2
9p13.2	*PAX5*	1,2,3,4,5,6,7,8,9,10	NM_016734.2
9q34.12	*ABL1**	2,3,11	NM_007313.2
9q34.13	*NUP214*	2.23.29	NM_005085.3
9q34.3	*NOTCH1*	2,14,25,26,28,31	NM_017617.3
10q23.31	*PTEN*	1,2,3,4,5,6,7,8,9	NM_000314.4
11p15.4	*LMO1*	1,2,4	NM_002315.2
11p13	*LMO2*	1,5,6	NM_005574.3
11p13	*CD44*	18	NM_000610.3
11p13	*SLC1A2*	11	NM_001195728.2
11p12	*RAG2*	1,2	NM_000536.3
12p13.2	*ETV6**	1,2,3,4,5,6,7,8	NM_001987.4
12q21.33	*BTG1*	1,2,downstream	NM_001731.2
13q14.2	*RB1*	5,14,19,24,26	NM_000321.2
14q32.33	*IGHM*	1,4	NG_001019.5
17p13.1	*TP53*	1,2,3,4,5,6,7,8,10,11	NM_000546.5
17q11.2	*NF1*	12,26,58	NM_001042492.2
17q11.2	*SUZ12*	7,10,15	NM_015355.2
17q12	*IKZF3*	4,5,7	NM_012481.4

18p11.21	*PTPN2*	1,2,3,8	NM_080422.2
21q11.2	*HSPA13*	4	NM_006948.4
21q11.2	*SAMSN1*	6	NM_001256370.1
21q21.1	*MIR99A*	NA	NR_136542
21q21.1	*BTG3*	5	NM_001130914.1
21q21.1	*TMPRSS15*	13	NM_002772.2
21q21.1	*NCAM2*	5	NM_004540.4
21q21.2	*MIR155*	NA	NR_001458
21q21.3	*ADAMTS5*	4	NM_007038.4
21q21.3	*APP*	3	NM_001204301.1
21q21.3	*CYYR1*	NA	NR_135515.1
21q21.3	*BACH1*	3	NM_001186.3
21q22.11	*TIAM1*	23	NM_003253.3
21q22.11	*OLIG2*	NA	NM_005806.3
21q22.11	*KCNE2*	2	NM_172201.1
21q22.12	*RUNX1**	2,4,6,7,8,9	NM_001754.4
21q22.13	*SIM2*	10	NM_005069.5
21q22.13	*HLCS*	7	NM_000411.7
21q22.13	*DYRK1A*	NA	NM_001396.4
21q22.13	*KCNJ6*	3	NM_002240.4
21q22.2	*ERG*	1,2,3,4,5,6,7,8,9,10,11,12	NM_001136154.1
21q22.2	*ETS2*	9	NM_001256295.1
21q22.2	*PSMG1*	6	NM_001261824.1
21q22.3	*TMPRSS2*	4	NM_001135099.1
21q22.3	*RIPK4*	3	NM_020639.2
21q22.3	*TFF1*	2	NM_003225.2
21q22.3	*ITGB2*	5	NM_001303238.1
21q22.3	*SLC19A1*	6	NM_194255.3
21q22.3	*COL6A2*	NA	NM_001849.3
21q22.3	*PRMT2*	4	NM_001242866.2
22q11.22	*VPREB1*	2	NM_007128.3
22q11.23	*IGLL1*	1	NM_020070.3
Xp22.33	*SHOX*	5,downstream	NM_000451.3
Xp22.33	*CRLF2*	1,2,3,4,5,6	NM_022148.3
Xp22.33	*CSF2RA*	1,2,3,4,5,6,7,8,9,10,11,13,14,15,16	NM_001161529.1
Xp22.33	*IL3RA*	1,2,3,6,7,8,9,12	NM_002183.3
Xp22.33	*P2RY8*	Upstream,1,2	NM_178129.4
Xq26.2	*PHF6*	1,3,7,10	NM_001015877

D007-X5-0220

Chromosome region	Gene	Exons	Reference
1p33	*TAL1*	1,3,5,6	NM_003189.5
1p33	*STIL*	1,2,6,12	NM_001048166.1

2q34	*IKZF2*	2,5,9	NM_016260.3
3p14.2	*FHIT***	4,8,9	NM_002012.4
3q13.2	*CD200*	3,4,6	NM_005944.7
3q13.2	*BTLA*	1,2,5	NM_181780.4
3q26.32	*TBL1XR1*	Upstream,1,2,7,15	NM_024665.7
4q25	*LEF1*	1,3,4,7,12	NM_016269.5
4q31.23	*NR3C2*	2,4,9	NM_000901.5
5q31.3	*NR3C1*	Upstream,1,2,5,8	NM_001018077.1
5q32	*PDGFRB*	9,10,11,12	NM_002609.4
5q33.3	*EBF1*	8,10,14,15,16	NM_001290360.3
5q31.2	*EGR1***	2	NM_001964.3
5q31.2	*CTNNA1***	6	NM_001903.5
5q33.1	*RPS14***	3	NM_005617.4
5q33.1	*SPARC***	7	NM_003118.4
5q35.3	*SQSTM1***	8	NM_003900.5
5q35.3	*FLT4***	12	NM_001354989
6q15	*CASP8AP2*	1,3,6,10	NM_012115.4
6q23.3	*MYB*	2,6,16	NM_001130173.2
7p12.2	*IKZF1*	Upstream,1,2,3,4,5,6,7,8 (two probes for each)	NM_006060.6
7p12.2	*IKZF1*	Alternative exon 4 (two probes)	NM_001291845.2
7q35, 7q34	*EPHA1***	3,8,12	NM_005232.5
7q36.1	*EZH2*	2,15,20	NM_004456.5
8q12.1	*TOX***	3,4,8	NM_014729.3
9p24.1	*JAK2***	5,7,10,20	NM_004972.4
9p21.3	*MLLT3*	2,5,7	NM_004529.4
9p21.3	*MTAP***	2,5,8	NM_002451.4
9p21.3	*CDKN2A*	Upstream,1,2,3,4 and downstream	NM_058195.4
9p21.3	*CDKN2B*	1,2	NM_078487.2
9p13.2	*PAX5*	1,2,3,4,5,6,7,8,9,10	NM_016734.3
9q34.12	*ABL1**	2,3,11	NM_007313.2
9q34.13	*NUP214*	2.23.29	NM_005085.4
9q34.3	*NOTCH1*	2,14,25,26,27,28,31	NM_017617.5
10q23.31	*PTEN*	1,2,3,4,5,6,7,8,9 (at least two probes for each)	NM_000314.8
10q25.2	*ADD3***	3,4,7	NM_016824.5
11p15.4	*LMO1*	1,2,4	NM_002315.3
11p13	*LMO2*	1,5,6	NM_005574.4
11p13	*CD44*	9	NM_000610.4
11p13	*SLC1A2*	10	NM_004171.4
11p12	*RAG2*	1,2	NM_000536.4
12p13.2	*ETV6**	1,2,3,4,5,6,7,8	NM_001987.5
12q21.33	*BTG1*	1,2,downstream	NM_001731.3
13q14.2	*RB1*	5,14,19,24,26	NM_000321.3
14q32.33	*IGHM*	1,4	NG_001019.6
15q14	*SPRED1***	3,4,7	NM_152594.3
16p13.3	*CREBBP***	6,8,31	NM_004380.3
16q22.1	*CTCF***	3,6,9	NM_006565.4
17p13.1	*TP53*	1,2a,2d,3,4b,5,6,7,8,10,11,downstream*	NM_000546.6

17q11.2	*NF1*	12,26,58	NM_001042492.3
17q11.2	*SUZ12*	7,10,15	NM_015355.4
17q12	*IKZF3*	4,5,7	NM_012481.5
18p11.21	*PTPN2*	1,2,3,8	NM_080422.2
21q11.2	*HSPA13*	2,4	NM_006948.5
21q11.2	*SAMSN1*	5	NM_022136.5
21q21.1	*MIR99A*	1	NR_136542
21q21.1	*BTG3*	5	NM_006806.5
21q21.1	*TMPRSS15*	13	NM_002772.3
21q21.1	*NCAM2*	5	NM_004540.5
21q21.2	*MIR155*	1	NR_001458
21q21.3	*ADAMTS5*	4	NM_007038.5
21q21.3	*APP*	3	NM_000484.4
21q21.3	*CYYR1*	2	NM_001320768.2
21q21.3	*BACH1*	3	NM_001186.4
21q22.11	*TIAM1*	23	NM_001353694.2
21q22.11	*OLIG2*	2	NM_005806.4
21q22.11	*KCNE2*	2	NM_172201.2
21q22.12	*RUNX1**	2,4,6,7,8,9	NM_001754.5
21q22.13	*SIM2*	10	NM_005069.6
21q22.13	*HLCS*	7	NM_000411.8
21q22.13	*DYRK1A*	4	NM_001396.5
21q22.13	*KCNJ6*	3	NM_002240.4
21q22.2	*ERG*	1,2,3,4,5,6,7,8,9,10,11,12	NM_001136154.1
21q22.2	*ETS2*	8	NM_001256295.2
21q22.2	*PSMG1*	6	NM_003720.4
21q22.3	*TMPRSS2*	4	NM_001135099.1
21q22.3	*RIPK4*	3	NM_020639.3
21q22.3	*TFF1*	2	NM_003225.3
21q22.3	*ITGB2*	5	NM_000211.5
21q22.3	*SLC19A1*	6	NM_194255.4
21q22.3	*COL6A2*	5	NM_001849.4
21q22.3	*PRMT2*	5	NM_206962.4
22q11.22	*VPREB1*	2	NM_007128.4
22q11.23	*IGLL1*	1	NM_020070.4
Xp22.33	*SHOX*	5,downstream	NM_000451.4
Xp22.33	*CRLF2*	1,2,3,4,5,6	NM_022148.4
Xp22.33	*CSF2RA*	1,2,3,4,5,6,7,8,9,10,11,13,14,15,16	NM_001161529.2
Xp22.33	*IL3RA*	1,2,3,6,7,8,9,12	NM_002183.4
Xp22.33	*P2RY8*	Upstream,1,2	NM_178129.5
Xp22.33	*AKAP17A***	3	NM_005088.3
Xp22.33	*ASMT***	6	NM_001171038.2
Xp22.33	*ZBED1***	2	NM_001171136.2
Xp22.33	*CD99***	10	NM_002414.5
Xp22.33	*GYG2***	12	NM_001184704.1
Xp21.1, Xp21.2	*DMD***	2,3,5,37,49,60	NM_004006.3
Xq26.2	*PHF6*	1,3,7,10	NM_001015877.2

* Copy number aberrations were validated by LSI (locus-specific identifier) *ETV6/RUNX1* and *BCR/ABL1* fusion extra-signal fluorescence *in situ* hybridization probe sets, routinely applied in the diagnostic workflow.
** Genes not covered by the D007-X2-0516 probemix which was used for analysing samples from patients #1-23, #84-104, #151-152, #154-160, #165, #169, #172-189.

Table S3. Characteristics of patients included in the validation cohort.

Patient ID	Sex	Diagnosis	Age at diagnosis (years)	Genetic subgroup*	Treatment protocol	TARGET ALL cohort
9906_071	Female	B-ALL	15.9	Hyperdiploidy	P9906	Phase 1
9906_028	Female	B-ALL	5.4	*TCF3-PBX1*	P9906	Phase 1
9906_260	Male	B-ALL	13.2	B-other**	P9906	Phase 1
9906_055	Female	B-ALL	12.2	B-other	P9906	Phase 1
9906_082	Female	B-ALL	7.6	B-other	P9906	Phase 1
9906_004	Female	B-ALL	2.3	B-other	P9906	Phase 1
9906_163	Female	B-ALL	11.0	*TCF3-PBX1*	P9906	Phase 1
9906_216	Female	B-ALL	11.9	B-other	P9906	Phase 1
9906_107	Male	B-ALL	1.9	B-other	P9906	Phase 1
9906_195	Male	B-ALL	11.8	B-other	P9906	Phase 1
9906_030	Female	B-ALL	14.5	B-other	P9906	Phase 1
9906_239	Female	B-ALL	14.0	B-other	P9906	Phase 1
9906_057	Male	B-ALL	13.0	B-other	P9906	Phase 1
9906_034	Male	B-ALL	13.1	B-other	P9906	Phase 1
9906_069	Male	B-ALL	15.2	B-other	P9906	Phase 1
9906_244	Male	B-ALL	14.5	Hyperdiploidy	P9906	Phase 1
9906_113	Male	B-ALL	16.8	B-other	P9906	Phase 1
9906_155	Female	B-ALL	11.2	B-other	P9906	Phase 1
9906_199	Male	B-ALL	2.4	B-other	P9906	Phase 1
9906_147	Female	B-ALL	16.8	B-other	P9906	Phase 1
9906_173	Male	B-ALL	17.4	B-other	P9906	Phase 1
9906_050	Male	B-ALL	5.6	B-other	P9906	Phase 1
9906_012	Male	B-ALL	12.8	B-other	P9906	Phase 1
9906_211	Male	B-ALL	2.9	B-other	P9906	Phase 1
9906_177	Male	B-ALL	14.6	B-other	P9906	Phase 1
9906_219	Male	B-ALL	14.3	B-other	P9906	Phase 1
9906_221	Male	B-ALL	11.8	B-other	P9906	Phase 1
9906_245	Male	B-ALL	2.7	Hyperdiploidy	P9906	Phase 1
9906_207	Male	B-ALL	13.2	Hyperdiploidy	P9906	Phase 1
9906_256	Male	B-ALL	15.2	B-other	P9906	Phase 1
9906_117	Female	B-ALL	7.6	B-other	P9906	Phase 1
9906_108	Male	B-ALL	2.4	B-other	P9906	Phase 1
9906_268	Male	B-ALL	17.3	B-other	P9906	Phase 1
9906_154	Female	B-ALL	1.7	B-other	P9906	Phase 1
9906_160	Male	B-ALL	14.3	B-other	P9906	Phase 1
9906_060	Male	B-ALL	17.9	B-other	P9906	Phase 1
9906_257	Female	B-ALL	13.3	B-other	P9906	Phase 1
9906_157	Male	B-ALL	16.7	B-other	P9906	Phase 1
9906_041	Male	B-ALL	14.0	KMT2Ar	P9906	Phase 1
9906_217	Male	B-ALL	17.7	B-other	P9906	Phase 1
9906_051	Male	B-ALL	1.1	KMT2Ar	P9906	Phase 1
9906_258	Male	B-ALL	7.9	B-other	P9906	Phase 1
9906_175	Female	B-ALL	2.0	B-other	P9906	Phase 1
9906_022	Male	B-ALL	9.4	Hyperdiploidy	P9906	Phase 1
9906_240	Male	B-ALL	13.9	Hyperdiploidy	P9906	Phase 1

9906_094	Male	B-ALL	13.3	Hyperdiploidy	P9906	Phase 1
9906_099	Female	B-ALL	5.8	Hyperdiploidy	P9906	Phase 1
9906_144	Female	B-ALL	3.5	Hyperdiploidy	P9906	Phase 1
9906_011	Male	B-ALL	12.1	Hyperdiploidy	P9906	Phase 1
9906_171	Female	B-ALL	3.6	Hyperdiploidy	P9906	Phase 1
9906_259	Male	B-ALL	13.5	B-other	P9906	Phase 1
9906_076	Female	B-ALL	3.6	Hyperdiploidy	P9906	Phase 1
9906_111	Male	B-ALL	13.0	B-other	P9906	Phase 1
9906_021	Male	B-ALL	2.4	B-other	P9906	Phase 1
9906_120	Male	B-ALL	3.9	B-other	P9906	Phase 1
9906_150	Male	B-ALL	12.6	B-other	P9906	Phase 1
9906_235	Female	B-ALL	2.9	B-other	P9906	Phase 1
9906_170	Male	B-ALL	16.1	B-other	P9906	Phase 1
9906_002	Male	B-ALL	4.9	*TCF3-PBX1*	P9906	Phase 1
9906_003	Male	B-ALL	14.6	*TCF3-PBX1*	P9906	Phase 1
9906_254	Female	B-ALL	11.2	B-other	P9906	Phase 1
9906_095	Female	B-ALL	2.8	KMT2Ar	P9906	Phase 1
9906_009	Female	B-ALL	2.9	B-other	P9906	Phase 1
9906_010	Male	B-ALL	14.7	B-other	P9906	Phase 1
9906_001	Male	B-ALL	6.9	B-other	P9906	Phase 1
9906_006	Male	B-ALL	1.3	KMT2Ar	P9906	Phase 1
9906_017	Female	B-ALL	10.7	*TCF3-PBX1*	P9906	Phase 1
9906_018	Male	B-ALL	2.1	Hyperdiploidy	P9906	Phase 1
9906_231	Female	B-ALL	17.6	B-other	P9906	Phase 1
9906_023	Female	B-ALL	11.2	B-other	P9906	Phase 1
9906_024	Male	B-ALL	5.4	B-other	P9906	Phase 1
9906_220	Female	B-ALL	9.1	KMT2Ar	P9906	Phase 1
9906_032	Male	B-ALL	2.2	KMT2Ar	P9906	Phase 1
9906_033	Male	B-ALL	17.2	B-other	P9906	Phase 1
9906_192	Male	B-ALL	5.6	B-other	P9906	Phase 1
9906_243	Female	B-ALL	3.9	B-other	P9906	Phase 1
9906_037	Male	B-ALL	1.4	B-other	P9906	Phase 1
9906_039	Male	B-ALL	17.3	B-other	P9906	Phase 1
9906_040	Female	B-ALL	16.4	B-other	P9906	Phase 1
9906_225	Male	B-ALL	16.6	B-other	P9906	Phase 1
9906_042	Male	B-ALL	15.1	B-other	P9906	Phase 1
9906_045	Male	B-ALL	14.5	B-other	P9906	Phase 1
9906_046	Female	B-ALL	4.2	*TCF3-PBX1*	P9906	Phase 1
9906_048	Male	B-ALL	3.4	B-other	P9906	Phase 1
9906_183	Male	B-ALL	17.0	B-other	P9906	Phase 1
9906_031	Male	B-ALL	16.6	B-other	P9906	Phase 1
9906_149	Male	B-ALL	17.1	B-other	P9906	Phase 1
9906_052	Male	B-ALL	15.1	B-other	P9906	Phase 1
9906_079	Female	B-ALL	15.5	*TCF3-PBX1*	P9906	Phase 1
9906_058	Male	B-ALL	17.8	*TCF3-PBX1*	P9906	Phase 1
9906_061	Female	B-ALL	16.2	B-other	P9906	Phase 1
9906_062	Male	B-ALL	16.6	B-other	P9906	Phase 1
9906_063	Male	B-ALL	16.1	*TCF3-PBX1*	P9906	Phase 1
9906_064	Male	B-ALL	17.8	B-other	P9906	Phase 1
9906_066	Male	B-ALL	15.9	B-other	P9906	Phase 1

9906_070	Male	B-ALL	16.7	B-other	P9906	Phase 1
9906_092	Female	B-ALL	6.0	B-other	P9906	Phase 1
9906_074	Male	B-ALL	1.6	KMT2Ar	P9906	Phase 1
9906_075	Male	B-ALL	12.2	*TCF3-PBX1*	P9906	Phase 1
9906_027	Male	B-ALL	11.7	B-other	P9906	Phase 1
9906_110	Female	B-ALL	4.0	B-other	P9906	Phase 1
9906_013	Male	B-ALL	14.4	B-other	P9906	Phase 1
9906_080	Male	B-ALL	1.8	B-other	P9906	Phase 1
9906_215	Male	B-ALL	17.2	B-other	P9906	Phase 1
9906_084	Male	B-ALL	2.7	B-other	P9906	Phase 1
9906_085	Female	B-ALL	1.9	B-other	P9906	Phase 1
9906_086	Male	B-ALL	11.4	B-other	P9906	Phase 1
9906_087	Male	B-ALL	15.2	B-other	P9906	Phase 1
9906_090	Male	B-ALL	16.0	B-other	P9906	Phase 1
9906_091	Female	B-ALL	2.1	KMT2Ar	P9906	Phase 1
9906_137	Male	B-ALL	13.1	KMT2Ar	P9906	Phase 1
9906_083	Male	B-ALL	6.9	*ETV6-RUNX1*	P9906	Phase 1
9906_096	Female	B-ALL	16.1	*TCF3-PBX1*	P9906	Phase 1
9906_097	Male	B-ALL	2.6	KMT2Ar	P9906	Phase 1
9906_098	Male	B-ALL	13.5	B-other	P9906	Phase 1
9906_100	Female	B-ALL	2.4	*TCF3-PBX1*	P9906	Phase 1
9906_102	Male	B-ALL	17.8	B-other	P9906	Phase 1
9906_106	Male	B-ALL	12.9	B-other	P9906	Phase 1
9906_065	Male	B-ALL	14.4	B-other	P9906	Phase 1
9906_109	Male	B-ALL	17.3	B-other	P9906	Phase 1
9906_112	Male	B-ALL	13.1	*TCF3-PBX1*	P9906	Phase 1
9906_114	Male	B-ALL	16.0	B-other	P9906	Phase 1
9906_115	Female	B-ALL	1.2	KMT2Ar	P9906	Phase 1
9906_116	Male	B-ALL	4.7	KMT2Ar	P9906	Phase 1
9906_118	Male	B-ALL	10.9	B-other	P9906	Phase 1
9906_119	Male	B-ALL	14.7	B-other	P9906	Phase 1
9906_121	Male	B-ALL	12.5	B-other	P9906	Phase 1
9906_123	Male	B-ALL	17.8	KMT2Ar	P9906	Phase 1
9906_174	Female	B-ALL	8.7	B-other	P9906	Phase 1
9906_073	Female	B-ALL	12.9	B-other	P9906	Phase 1
9906_133	Female	B-ALL	14.3	B-other	P9906	Phase 1
9906_135	Male	B-ALL	15.3	B-other	P9906	Phase 1
9906_136	Male	B-ALL	17.3	B-other	P9906	Phase 1
9906_138	Male	B-ALL	14.3	B-other	P9906	Phase 1
9906_142	Female	B-ALL	10.8	KMT2Ar	P9906	Phase 1
9906_145	Male	B-ALL	3.2	B-other	P9906	Phase 1
9906_146	Male	B-ALL	9.8	B-other	P9906	Phase 1
9906_176	Male	B-ALL	1.4	B-other	P9906	Phase 1
9906_152	Female	B-ALL	3.3	*TCF3-PBX1*	P9906	Phase 1
9906_153	Female	B-ALL	17.4	B-other	P9906	Phase 1
9906_251	Female	B-ALL	2.0	Hyperdiploidy	P9906	Phase 1
9906_156	Female	B-ALL	2.8	*TCF3-PBX1*	P9906	Phase 1
9906_158	Female	B-ALL	1.5	B-other	P9906	Phase 1
9906_159	Female	B-ALL	2.8	*TCF3-PBX1*	P9906	Phase 1
9906_005	Female	B-ALL	4.8	B-other	P9906	Phase 1

9906_161	Male	B-ALL	14.2	B-other	P9906	Phase 1
9906_124	Male	B-ALL	12.8	B-other	P9906	Phase 1
9906_165	Male	B-ALL	12.7	B-other	P9906	Phase 1
9906_166	Male	B-ALL	15.4	*TCF3-PBX1*	P9906	Phase 1
9906_167	Male	B-ALL	4.0	B-other	P9906	Phase 1
9906_168	Male	B-ALL	13.6	B-other	P9906	Phase 1
9906_132	Male	B-ALL	1.6	Hyperdiploidy	P9906	Phase 1
9906_262	Female	B-ALL	16.7	B-other	P9906	Phase 1
9906_172	Male	B-ALL	14.0	B-other	P9906	Phase 1
9906_049	Female	B-ALL	16.4	B-other	P9906	Phase 1
9906_236	Male	B-ALL	3.7	*TCF3-PBX1*	P9906	Phase 1
9906_179	Male	B-ALL	16.5	B-other	P9906	Phase 1
9906_180	Male	B-ALL	1.8	B-other	P9906	Phase 1
9906_182	Male	B-ALL	12.6	B-other	P9906	Phase 1
9906_184	Female	B-ALL	2.6	B-other	P9906	Phase 1
9906_185	Male	B-ALL	1.0	B-other	P9906	Phase 1
9906_187	Male	B-ALL	13.5	*TCF3-PBX1*	P9906	Phase 1
9906_188	Male	B-ALL	14.7	B-other	P9906	Phase 1
9906_189	Male	B-ALL	12.6	B-other	P9906	Phase 1
9906_190	Male	B-ALL	14.1	B-other	P9906	Phase 1
9906_016	Male	B-ALL	11.0	B-other	P9906	Phase 1
9906_196	Male	B-ALL	14.2	B-other	P9906	Phase 1
9906_198	Female	B-ALL	2.7	*TCF3-PBX1*	P9906	Phase 1
9906_129	Male	B-ALL	2.2	Hyperdiploidy	P9906	Phase 1
9906_200	Male	B-ALL	16.0	B-other	P9906	Phase 1
9906_201	Male	B-ALL	2.0	B-other	P9906	Phase 1
9906_202	Male	B-ALL	12.1	*TCF3-PBX1*	P9906	Phase 1
9906_203	Male	B-ALL	4.8	*TCF3-PBX1*	P9906	Phase 1
9906_205	Male	B-ALL	15.2	B-other	P9906	Phase 1
9906_206	Male	B-ALL	15.1	Hyperdiploidy	P9906	Phase 1
9906_209	Female	B-ALL	16.5	Hyperdiploidy	P9906	Phase 1
9906_213	Male	B-ALL	5.2	Hyperdiploidy	P9906	Phase 1
9906_093	Female	B-ALL	11.8	B-other	P9906	Phase 1
9906_218	Male	B-ALL	13.8	*TCF3-PBX1*	P9906	Phase 1
9906_128	Female	B-ALL	1.1	KMT2Ar	P9906	Phase 1
9906_222	Female	B-ALL	17.6	B-other	P9906	Phase 1
9906_224	Male	B-ALL	1.2	*ETV6-RUNX1*	P9906	Phase 1
9906_019	Female	B-ALL	1.2	B-other	P9906	Phase 1
9906_227	Female	B-ALL	14.9	KMT2Ar	P9906	Phase 1
9906_228	Male	B-ALL	17.0	B-other	P9906	Phase 1
9906_229	Female	B-ALL	12.5	KMT2Ar	P9906	Phase 1
9906_230	Male	B-ALL	17.3	B-other	P9906	Phase 1
9906_101	Female	B-ALL	16.2	B-other	P9906	Phase 1
9906_232	Male	B-ALL	16.3	B-other	P9906	Phase 1
9906_020	Male	B-ALL	13.9	B-other	P9906	Phase 1
9906_234	Male	B-ALL	6.0	B-other	P9906	Phase 1
9906_233	Male	B-ALL	7.9	B-other	P9906	Phase 1
9906_014	Female	B-ALL	12.9	iAMP21***	P9906	Phase 1
9906_241	Male	B-ALL	16.8	B-other	P9906	Phase 1
9906_242	Male	B-ALL	14.8	B-other	P9906	Phase 1

9906_122	Male	B-ALL	5.2	Hyperdiploidy	P9906	Phase 1
9906_246	Male	B-ALL	14.2	B-other	P9906	Phase 1
9906_247	Male	B-ALL	2.3	KMT2Ar	P9906	Phase 1
9906_249	Male	B-ALL	2.5	Hyperdiploidy	P9906	Phase 1
9906_250	Male	B-ALL	17.3	B-other	P9906	Phase 1
9906_252	Male	B-ALL	15.1	B-other	P9906	Phase 1
9906_143	Female	B-ALL	13.3	iAMP21	P9906	Phase 1
9906_255	Male	B-ALL	17.3	B-other	P9906	Phase 1
9906_261	Male	B-ALL	15.5	KMT2Ar	P9906	Phase 1
9906_263	Male	B-ALL	14.2	B-other	P9906	Phase 1
9906_265	Male	B-ALL	13.6	B-other	P9906	Phase 1
9906_267	Male	B-ALL	16.1	B-other	P9906	Phase 1
9906_271	Male	B-ALL	15.8	B-other	P9906	Phase 1
9906_272	Female	B-ALL	14.5	*TCF3-PBX1*	P9906	Phase 1
TARGET-10-PANSIZ-09A-01D	Male	B-ALL	4.5	Hyperdiploidy	AALL0331	Phase 2
TARGET-10-PANSTA-09A-01D	Female	B-ALL	2.4	B-other	AALL0331	Phase 2
TARGET-10-PANSYA-09A-01D	Female	B-ALL	4.0	*ETV6-RUNX1*	AALL0331	Phase 2
TARGET-10-PANTCR-09A-01D	Male	B-ALL	5.6	*ETV6-RUNX1*	AALL0331	Phase 2
TARGET-10-PANTLF-03A-01D	Female	B-ALL	1.6	KMT2Ar	AALL0331	Phase 2
TARGET-10-PANTRY-09A-01D	Male	B-ALL	4.5	*ETV6-RUNX1*	AALL0331	Phase 2
TARGET-10-PANTTB-09A-01D	Male	B-ALL	7.4	Hyperdiploidy	AALL0331	Phase 2
TARGET-10-PANTUZ-09A-01D	Male	B-ALL	2.6	Hyperdiploidy	AALL0331	Phase 2
TARGET-10-PANTVC-09A-01D	Male	B-ALL	1.8	B-other	AALL0331	Phase 2
TARGET-10-PANTWC-09A-01D	Male	B-ALL	4.2	B-other	AALL0331	Phase 2
TARGET-10-PANTXA-09A-01D	Female	B-ALL	3.7	Hyperdiploidy	AALL0331	Phase 2
TARGET-10-PANTYP-09A-01D	Female	B-ALL	3.1	Hyperdiploidy	AALL0331	Phase 2
TARGET-10-PANTZE-09B-01D	Female	B-ALL	3.5	*ETV6-RUNX1*	AALL0331	Phase 2
TARGET-10-PANUKF-09A-01D	Male	B-ALL	3.7	*ETV6-RUNX1*	AALL0331	Phase 2
TARGET-10-PANURR-09A-01D	Female	B-ALL	3.0	Hyperdiploidy	AALL0331	Phase 2
TARGET-10-PANUSL-09A-01D	Male	B-ALL	2.7	Hyperdiploidy	AALL0331	Phase 2
TARGET-10-PANUXU-09A-01D	Female	B-ALL	7.0	*ETV6-RUNX1*	AALL0331	Phase 2
TARGET-10-PANUYZ-09A-01D	Male	B-ALL	1.9	B-other	AALL0331	Phase 2
TARGET-10-PANVCM-09A-01D	Male	B-ALL	1.6	Hyperdiploidy	AALL0331	Phase 2
TARGET-10-PANVDH-09A-01D	Female	B-ALL	5.6	*ETV6-RUNX1*	AALL0331	Phase 2
TARGET-10-PANVDV-09A-01D	Male	B-ALL	7.5	B-other	AALL0331	Phase 2
TARGET-10-PANVFF-09A-01D	Male	B-ALL	5.0	B-other	AALL0331	Phase 2
TARGET-10-PANVIB-09A-01D	Male	B-ALL	2.8	Hyperdiploidy	AALL0331	Phase 2
TARGET-10-PANVIC-03A-01D	Female	B-ALL	7.6	iAMP21	AALL0331	Phase 2
TARGET-10-PANVJI-09A-01D	Female	B-ALL	6.2	B-other	AALL0331	Phase 2
TARGET-10-PANVKF-09A-01D	Female	B-ALL	6.7	*ETV6-RUNX1*	AALL0331	Phase 2
TARGET-10-PANVKH-09A-01D	Female	B-ALL	4.4	Hyperdiploidy	AALL0331	Phase 2
TARGET-10-PANVMT-09A-01D	Female	B-ALL	9.2	*TCF3-PBX1*	AALL0331	Phase 2
TARGET-10-PANVTB-09A-01D	Male	B-ALL	2.4	B-other	AALL0331	Phase 2
TARGET-10-PANVXF-09A-01D	Female	B-ALL	4.1	*TCF3-PBX1*	AALL0331	Phase 2
TARGET-10-PANWDL-09A-01D	Male	B-ALL	3.9	B-other	AALL0331	Phase 2
TARGET-10-PANWDN-09A-01D	Male	B-ALL	9.5	B-other	AALL0331	Phase 2
TARGET-10-PANWDS-09A-01D	Male	B-ALL	5.9	B-other	AALL0331	Phase 2
TARGET-10-PANWEI-09A-01D	Female	B-ALL	4.4	B-other	AALL0331	Phase 2
TARGET-10-PANWGG-09A-01D	Female	B-ALL	3.7	*ETV6-RUNX1*	AALL0331	Phase 2
TARGET-10-PANWIM-09A-01D	Female	B-ALL	2.8	*ETV6-RUNX1*	AALL0331	Phase 2

TARGET-10-PANWJH-09A-01D	Female	B-ALL	7.1	*ETV6-RUNX1*	AALL0331	Phase 2
TARGET-10-PANWJR-09A-01D	Female	B-ALL	5.3	*ETV6-RUNX1*	AALL0331	Phase 2
TARGET-10-PANWLH-09A-01D	Male	B-ALL	2.3	Hyperdiploidy	AALL0331	Phase 2
TARGET-10-PANWWG-09A-01D	Female	B-ALL	3.6	Hyperdiploidy	AALL0331	Phase 2
TARGET-10-PANWZG-09A-01D	Male	B-ALL	4.3	*ETV6-RUNX1*	AALL0331	Phase 2
TARGET-10-PANXAM-09A-01D	Female	B-ALL	4.8	*ETV6-RUNX1*	AALL0331	Phase 2
TARGET-10-PANXCX-09A-01D	Male	B-ALL	2.5	*ETV6-RUNX1*	AALL0331	Phase 2
TARGET-10-PANXEE-09A-01D	Male	B-ALL	2.6	*ETV6-RUNX1*	AALL0331	Phase 2
TARGET-10-PANXGD-09A-01D	Male	B-ALL	8.2	iAMP21	AALL0331	Phase 2
TARGET-10-PANXGM-09A-01D	Male	B-ALL	9.6	Hyperdiploidy	AALL0331	Phase 2
TARGET-10-PANXLC-09A-01D	Female	B-ALL	2.1	Hyperdiploidy	AALL0331	Phase 2
TARGET-10-PANXLP-09A-01D	Male	B-ALL	6.0	Hyperdiploidy	AALL0331	Phase 2
TARGET-10-PANXLR-09A-01D	Female	B-ALL	2.0	*ETV6-RUNX1*	AALL0331	Phase 2
TARGET-10-PANXSF-09A-01D	Male	B-ALL	2.1	Hyperdiploidy	AALL0331	Phase 2
TARGET-10-PANXTP-09A-01D	Male	B-ALL	3.0	Hyperdiploidy	AALL0331	Phase 2
TARGET-10-PANYHB-03A-01D	Male	B-ALL	2.3	Hyperdiploidy	AALL0331	Phase 2
TARGET-10-PANYJV-09A-01D	Female	B-ALL	5.8	Hyperdiploidy	AALL0331	Phase 2
TARGET-10-PANYXD-09A-01D	Male	B-ALL	4.3	Hyperdiploidy	AALL0331	Phase 2
TARGET-10-PANYXR-09A-01D	Female	B-ALL	1.5	Hyperdiploidy	AALL0331	Phase 2
TARGET-10-PANYZE-09A-01D	Female	B-ALL	4.1	B-other	AALL0331	Phase 2
TARGET-10-PANZBR-09A-01D	Male	B-ALL	1.2	*ETV6-RUNX1*	AALL0331	Phase 2
TARGET-10-PANZCF-09A-01D	Male	B-ALL	8.5	iAMP21	AALL0331	Phase 2
TARGET-10-PANZEG-03A-01D	Female	B-ALL	2.3	Hyperdiploidy	AALL0331	Phase 2
TARGET-10-PANZFN-09A-01D	Female	B-ALL	6.2	*ETV6-RUNX1*	AALL0331	Phase 2
TARGET-10-PANZGN-09A-01D	Male	B-ALL	1.7	*ETV6-RUNX1*	AALL0331	Phase 2
TARGET-10-PANZPU-09A-01D	Male	B-ALL	4.7	Hyperdiploidy	AALL0331	Phase 2
TARGET-10-PANZSE-09A-01D	Female	B-ALL	3.2	B-other	AALL0331	Phase 2
TARGET-10-PANZUI-09A-01D	Female	B-ALL	8.6	B-other	AALL0331	Phase 2
TARGET-10-PANZYY-09A-01D	Male	B-ALL	1.5	Hyperdiploidy	AALL0331	Phase 2
TARGET-10-PANZZI-09A-01D	Male	B-ALL	5.8	*ETV6-RUNX1*	AALL0331	Phase 2
TARGET-10-PAPADT-09A-01D	Male	B-ALL	1.6	Hyperdiploidy	AALL0331	Phase 2
TARGET-10-PAPAGB-09A-01D	Female	B-ALL	8.0	Hyperdiploidy	AALL0331	Phase 2
TARGET-10-PAPAGV-09A-01D	Female	B-ALL	3.0	*ETV6-RUNX1*	AALL0331	Phase 2
TARGET-10-PAPAKJ-09A-01D	Male	B-ALL	3.4	*ETV6-RUNX1*	AALL0331	Phase 2
TARGET-10-PAPAMA-09A-01D	Male	B-ALL	6.9	B-other	AALL0331	Phase 2
TARGET-10-PAPANB-09A-01D	Male	B-ALL	6.0	Hyperdiploidy	AALL0331	Phase 2
TARGET-10-PAPAPC-09A-01D	Female	B-ALL	2.4	Hyperdiploidy	AALL0331	Phase 2
TARGET-10-PAPAPX-09A-01D	Male	B-ALL	4.8	*ETV6-RUNX1*	AALL0331	Phase 2
TARGET-10-PAPART-09A-01D	Female	B-ALL	2.2	*ETV6-RUNX1*	AALL0331	Phase 2
TARGET-10-PAPAVR-09A-01D	Female	B-ALL	3.6	Hyperdiploidy	AALL0331	Phase 2
TARGET-10-PAPAXH-09A-01D	Female	B-ALL	2.7	B-other	AALL0331	Phase 2
TARGET-10-PAPAZA-09A-01D	Female	B-ALL	8.1	*ETV6-RUNX1*	AALL0331	Phase 2
TARGET-10-PAPAZD-09A-01D	Male	B-ALL	7.0	B-other	AALL0331	Phase 2
TARGET-10-PAPBAI-09A-01D	Male	B-ALL	2.6	Hyperdiploidy	AALL0331	Phase 2
TARGET-10-PAPBAN-09A-01D	Female	B-ALL	5.0	Hyperdiploidy	AALL0331	Phase 2
TARGET-10-PAPBLU-09A-01D	Male	B-ALL	5.1	*ETV6-RUNX1*	AALL0331	Phase 2
TARGET-10-PAPBZW-09A-01D	Male	B-ALL	3.1	B-other	AALL0331	Phase 2
TARGET-10-PAPCED-09A-01D	Female	B-ALL	4.4	*ETV6-RUNX1*	AALL0331	Phase 2
TARGET-10-PAPCJR-09A-01D	Male	B-ALL	2.8	*ETV6-RUNX1*	AALL0331	Phase 2
TARGET-10-PAPCNP-09A-01D	Male	B-ALL	9.6	B-other	AALL0331	Phase 2

TARGET-10-PAPCPB-09A-01D	Male	B-ALL	4.2	*ETV6-RUNX1*	AALL0331	Phase 2
TARGET-10-PAPCRU-03A-01D	Male	B-ALL	6.7	Hyperdiploidy	AALL0331	Phase 2
TARGET-10-PAPCVI-09A-01D	Female	B-ALL	4.2	*ETV6-RUNX1*	AALL0331	Phase 2
TARGET-10-PAPCVR-09A-01D	Female	B-ALL	2.8	*ETV6-RUNX1*	AALL0331	Phase 2
TARGET-10-PAPDCS-09A-01D	Male	B-ALL	5.4	*ETV6-RUNX1*	AALL0331	Phase 2
TARGET-10-PAPDDA-09A-01D	Male	B-ALL	4.6	Hyperdiploidy	AALL0331	Phase 2
TARGET-10-PAPDFS-09A-01D	Female	B-ALL	4.1	Hyperdiploidy	AALL0331	Phase 2
TARGET-10-PAPDHA-09A-01D	Male	B-ALL	2.1	Hyperdiploidy	AALL0331	Phase 2
TARGET-10-PAPDKD-09A-01D	Male	B-ALL	2.3	*ETV6-RUNX1*	AALL0331	Phase 2
TARGET-10-PAPDKR-09A-01D	Female	B-ALL	1.7	Hyperdiploidy	AALL0331	Phase 2
TARGET-10-PAPDLN-09A-01D	Male	B-ALL	2.2	B-other	AALL0331	Phase 2
TARGET-10-PAPDMU-09A-01D	Male	B-ALL	5.8	B-other	AALL0331	Phase 2
TARGET-10-PAPDSW-09A-01D	Male	B-ALL	3.6	*ETV6-RUNX1*	AALL0331	Phase 2
TARGET-10-PAPDUV-09A-01D	Female	B-ALL	5.4	iAMP21	AALL0331	Phase 2
TARGET-10-PAPDUX-09A-01D	Female	B-ALL	3.7	B-other	AALL0331	Phase 2
TARGET-10-PAPDVG-09A-01D	Female	B-ALL	7.3	*BCR-ABL1*	AALL0331	Phase 2
TARGET-10-PAPEJA-09A-01D	Male	B-ALL	3.3	*ETV6-RUNX1*	AALL0331	Phase 2
TARGET-10-PAPEJL-09A-01D	Female	B-ALL	9.5	*ETV6-RUNX1*	AALL0331	Phase 2
TARGET-10-PAPEJM-09A-01D	Female	B-ALL	3.7	*ETV6-RUNX1*	AALL0331	Phase 2
TARGET-10-PAPEJN-09A-01D	Male	B-ALL	3.1	*ETV6-RUNX1*	AALL0331	Phase 2
TARGET-10-PAPERN-09A-01D	Male	B-ALL	3.8	Hypodiploidy	AALL0331	Phase 2
TARGET-10-PAPERU-09A-01D	Female	B-ALL	5.8	*ETV6-RUNX1*	AALL0331	Phase 2
TARGET-10-PAPERW-09A-01D	Female	B-ALL	5.5	Hyperdiploidy	AALL0331	Phase 2
TARGET-10-PAPESY-09A-01D	Female	B-ALL	2.2	Hyperdiploidy	AALL0331	Phase 2
TARGET-10-PAPESZ-09A-01D	Male	B-ALL	3.9	*ETV6-RUNX1*	AALL0331	Phase 2
TARGET-10-PAPETC-09A-01D	Male	B-ALL	4.7	*ETV6-RUNX1*	AALL0331	Phase 2
TARGET-10-PAPEZR-09A-01D	Male	B-ALL	1.8	*ETV6-RUNX1*	AALL0331	Phase 2
TARGET-10-PAPFAT-09A-01D	Female	B-ALL	6.0	B-other	AALL0331	Phase 2
TARGET-10-PAPFBR-09A-01D	Male	B-ALL	2.9	*ETV6-RUNX1*	AALL0331	Phase 2
TARGET-10-PAPFBX-09A-01D	Female	B-ALL	4.4	Hyperdiploidy	AALL0331	Phase 2
TARGET-10-PAPFFW-09A-01D	Male	B-ALL	3.1	*ETV6-RUNX1*	AALL0331	Phase 2
TARGET-10-PAPFHH-09A-01D	Female	B-ALL	4.9	Hyperdiploidy	AALL0331	Phase 2
TARGET-10-PAPFHR-09A-01D	Male	B-ALL	2.4	*ETV6-RUNX1*	AALL0331	Phase 2
TARGET-10-PAPFHX-09A-01D	Female	B-ALL	9.4	B-other	AALL0331	Phase 2
TARGET-10-PAPFKA-09A-01D	Female	B-ALL	3.6	Hyperdiploidy	AALL0331	Phase 2
TARGET-10-PAPFNV-09A-01D	Female	B-ALL	4.5	*ETV6-RUNX1*	AALL0331	Phase 2
TARGET-10-PAPFPZ-09A-01D	Female	B-ALL	6.5	*ETV6-RUNX1*	AALL0331	Phase 2
TARGET-10-PAPFTJ-09A-01D	Female	B-ALL	5.8	*ETV6-RUNX1*	AALL0331	Phase 2
TARGET-10-PAPFUF-09A-01D	Female	B-ALL	7.5	*ETV6-RUNX1*	AALL0331	Phase 2
TARGET-10-PAPFZF-09A-01D	Female	B-ALL	3.6	B-other	AALL0331	Phase 2
TARGET-10-PAPFZL-09A-01D	Female	B-ALL	2.5	Hyperdiploidy	AALL0331	Phase 2
TARGET-10-PAPFZX-09A-01D	Female	B-ALL	3.2	*ETV6-RUNX1*	AALL0331	Phase 2
TARGET-10-PAPGEE-09A-01D	Female	B-ALL	9.0	*ETV6-RUNX1*	AALL0331	Phase 2
TARGET-10-PAPGFD-09A-01D	Male	B-ALL	3.3	B-other	AALL0331	Phase 2
TARGET-10-PAPGKP-09A-01D	Female	B-ALL	6.3	B-other	AALL0331	Phase 2
TARGET-10-PAPGLS-09A-01D	Male	B-ALL	9.8	*ETV6-RUNX1*	AALL0331	Phase 2
TARGET-10-PAPGMT-09A-01D	Female	B-ALL	3.8	Hyperdiploidy	AALL0331	Phase 2
TARGET-10-PAPGWN-09A-01D	Male	B-ALL	7.8	B-other	AALL0331	Phase 2
TARGET-10-PAPHAM-09A-01D	Male	B-ALL	2.7	*ETV6-RUNX1*	AALL0331	Phase 2
TARGET-10-PAPHBW-09A-01D	Male	B-ALL	6.5	*TCF3-PBX1*	AALL0331	Phase 2

TARGET-10-PAPHCA-09A-01D	Female	B-ALL	2.4	Hyperdiploidy	AALL0331	Phase 2
TARGET-10-PAPHDN-09A-01D	Female	B-ALL	4.7	*ETV6-RUNX1*	AALL0331	Phase 2
TARGET-10-PAPHDX-09A-01D	Female	B-ALL	2.2	Hyperdiploidy	AALL0331	Phase 2
TARGET-10-PAPHED-09A-01D	Female	B-ALL	2.1	*ETV6-RUNX1*	AALL0331	Phase 2
TARGET-10-PAPHHP-09A-01D	Male	B-ALL	5.5	Hyperdiploidy	AALL0331	Phase 2
TARGET-10-PAPHJF-09A-01D	Female	B-ALL	6.9	*TCF3-PBX1*	AALL0331	Phase 2
TARGET-10-PAPHLH-09A-01D	Female	B-ALL	4.6	B-other	AALL0331	Phase 2
TARGET-10-PAPHNM-09A-01D	Female	B-ALL	1.5	B-other	AALL0331	Phase 2
TARGET-10-PAPHNR-09A-01D	Female	B-ALL	9.1	Hyperdiploidy	AALL0331	Phase 2
TARGET-10-PAPHRV-09A-01D	Female	B-ALL	3.4	*ETV6-RUNX1*	AALL0331	Phase 2
TARGET-10-PAPHWE-09A-01D	Female	B-ALL	4.3	iAMP21	AALL0331	Phase 2
TARGET-10-PAPHYV-09A-01D	Male	B-ALL	2.7	*ETV6-RUNX1*	AALL0331	Phase 2
TARGET-10-PAPICC-09A-01D	Male	B-ALL	4.1	Hyperdiploidy	AALL0331	Phase 2
TARGET-10-PAPIEW-09A-01D	Male	B-ALL	5.6	Hyperdiploidy	AALL0331	Phase 2
TARGET-10-PAPIGD-09A-01D	Female	B-ALL	5.1	*ETV6-RUNX1*	AALL0331	Phase 2
TARGET-10-PAPIGV-09A-01D	Male	B-ALL	3.8	*ETV6-RUNX1*	AALL0331	Phase 2
TARGET-10-PAPIHH-09A-01D	Male	B-ALL	4.8	*ETV6-RUNX1*	AALL0331	Phase 2
TARGET-10-PAPIHT-09A-01D	Female	B-ALL	2.6	Hyperdiploidy	AALL0331	Phase 2
TARGET-10-PAPIHU-09A-01D	Male	B-ALL	3.5	Hyperdiploidy	AALL0331	Phase 2
TARGET-10-PAPIIB-09A-01D	Male	B-ALL	4.2	*ETV6-RUNX1*	AALL0331	Phase 2
TARGET-10-PAPIIX-09A-01D	Male	B-ALL	6.4	*ETV6-RUNX1*	AALL0331	Phase 2
TARGET-10-PAPIJD-09A-01D	Male	B-ALL	3.4	Hyperdiploidy	AALL0331	Phase 2
TARGET-10-PAPILF-09A-01D	Male	B-ALL	4.5	Hyperdiploidy	AALL0331	Phase 2
TARGET-10-PAPIPG-09A-01D	Male	B-ALL	4.3	Hyperdiploidy	AALL0331	Phase 2
TARGET-10-PAPZPZ-09A-01D	Female	B-ALL	3.4	Hyperdiploidy	AALL0331	Phase 2
TARGET-10-PAPZRB-09A-01D	Male	B-ALL	2.5	B-other	AALL0331	Phase 2
TARGET-10-PAPZTD-09A-01D	Male	B-ALL	8.2	B-other	AALL0331	Phase 2
TARGET-10-PAPZTL-09A-01D	Male	B-ALL	7.6	*ETV6-RUNX1*	AALL0331	Phase 2
TARGET-10-PAPZTS-09A-01D	Male	B-ALL	6.0	*ETV6-RUNX1*	AALL0331	Phase 2
TARGET-10-PAPZUE-03A-01D	Male	B-ALL	4.0	Hyperdiploidy	AALL0331	Phase 2
TARGET-10-PAPZUW-09A-01D	Male	B-ALL	3.6	*ETV6-RUNX1*	AALL0331	Phase 2
TARGET-10-PAPZWP-09A-01D	Female	B-ALL	2.8	B-other	AALL0331	Phase 2
TARGET-10-PAPZXI-09A-01D	Female	B-ALL	3.0	Hyperdiploidy	AALL0331	Phase 2
TARGET-10-PAPZZJ-09A-01D	Male	B-ALL	1.9	Hyperdiploidy	AALL0331	Phase 2
TARGET-10-PARABL-09A-01D	Female	B-ALL	6.0	*ETV6-RUNX1*	AALL0331	Phase 2
TARGET-10-PARABU-09A-01D	Male	B-ALL	9.5	B-other	AALL0331	Phase 2
TARGET-10-PARAFH-09A-01D	Male	B-ALL	4.2	*ETV6-RUNX1*	AALL0331	Phase 2
TARGET-10-PARAFI-09A-01D	Male	B-ALL	5.2	Hyperdiploidy	AALL0331	Phase 2
TARGET-10-PARAGW-09A-01D	Male	B-ALL	3.8	*ETV6-RUNX1*	AALL0331	Phase 2
TARGET-10-PARAJY-09A-01D	Male	B-ALL	2.4	Hyperdiploidy	AALL0331	Phase 2
TARGET-10-PARANN-09A-01D	Female	B-ALL	1.5	Hyperdiploidy	AALL0331	Phase 2
TARGET-10-PARASC-09A-01D	Female	B-ALL	5.5	Hyperdiploidy	AALL0331	Phase 2
TARGET-10-PARASN-09A-01D	Female	B-ALL	9.9	B-other	AALL0331	Phase 2
TARGET-10-PARATY-09A-01D	Male	B-ALL	2.6	Hyperdiploidy	AALL0331	Phase 2
TARGET-10-PARAUE-09A-01D	Male	B-ALL	3.4	B-other	AALL0331	Phase 2
TARGET-10-PARAXH-09A-01D	Female	B-ALL	3.8	*ETV6-RUNX1*	AALL0331	Phase 2
TARGET-10-PARAZN-09A-01D	Female	B-ALL	2.5	B-other	AALL0331	Phase 2
TARGET-10-PARBBV-09A-01D	Female	B-ALL	3.3	Hyperdiploidy	AALL0331	Phase 2
TARGET-10-PARBCW-09A-01D	Female	B-ALL	6.2	B-other	AALL0331	Phase 2
TARGET-10-PARBDP-09A-01D	Male	B-ALL	3.9	B-other	AALL0331	Phase 2

TARGET-10-PARBGG-09A-01D	Male	B-ALL	1.9	B-other	AALL0331	Phase 2
TARGET-10-PARBKG-09A-01D	Female	B-ALL	1.8	Hyperdiploidy	AALL0331	Phase 2
TARGET-10-PARBKP-09A-01D	Male	B-ALL	5.8	B-other	AALL0331	Phase 2
TARGET-10-PARBLL-09A-01D	Male	B-ALL	5.4	Hyperdiploidy	AALL0331	Phase 2
TARGET-10-PARBLS-09A-01D	Female	B-ALL	2.8	B-other	AALL0331	Phase 2
TARGET-10-PARBND-09A-01D	Male	B-ALL	4.2	Hyperdiploidy	AALL0331	Phase 2
TARGET-10-PARBNY-09A-01D	Male	B-ALL	2.7	Hyperdiploidy	AALL0331	Phase 2
TARGET-10-PARBPX-09A-01D	Female	B-ALL	6.2	*ETV6-RUNX1*	AALL0331	Phase 2
TARGET-10-PARBRM-09A-01D	Female	B-ALL	17.4	Hyperdiploidy	AALL0232	Phase 2
TARGET-10-PARBRV-09A-01D	Female	B-ALL	9.0	*ETV6-RUNX1*	AALL0331	Phase 2
TARGET-10-PARBSP-09A-01D	Male	B-ALL	3.4	*ETV6-RUNX1*	AALL0331	Phase 2
TARGET-10-PARBSW-09A-01D	Female	B-ALL	3.3	*ETV6-RUNX1*	AALL0331	Phase 2
TARGET-10-PARBTA-09A-01D	Male	B-ALL	7.3	Hyperdiploidy	AALL0331	Phase 2
TARGET-10-PARBWN-09A-01D	Male	B-ALL	14.4	*TCF3-PBX1*	AALL0232	Phase 2
TARGET-10-PARBXX-09A-01D	Male	B-ALL	5.6	B-other	AALL0331	Phase 2
TARGET-10-PARBYS-09A-01D	Female	B-ALL	2.4	B-other	AALL0331	Phase 2
TARGET-10-PARBYU-09A-01D	Female	B-ALL	4.1	Hyperdiploidy	AALL0331	Phase 2
TARGET-10-PARBZT-09A-01D	Female	B-ALL	10.2	iAMP21	AALL0232	Phase 2
TARGET-10-PARCAX-09A-01D	Male	B-ALL	4.2	*ETV6-RUNX1*	AALL0331	Phase 2
TARGET-10-PARCBE-09A-01D	Female	B-ALL	5.0	*ETV6-RUNX1*	AALL0331	Phase 2
TARGET-10-PARCCM-09A-01D	Male	B-ALL	7.1	Hyperdiploidy	AALL0331	Phase 2
TARGET-10-PARCDS-09A-01D	Female	B-ALL	13.4	B-other	AALL0232	Phase 2
TARGET-10-PARCDV-09A-01D	Male	B-ALL	4.4	*TCF3-PBX1*	AALL0232	Phase 2
TARGET-10-PARCDX-09A-01D	Female	B-ALL	4.0	B-other	AALL0331	Phase 2
TARGET-10-PARCDZ-09A-01D	Female	B-ALL	5.4	B-other	AALL0331	Phase 2
TARGET-10-PARCGU-09A-01D	Male	B-ALL	2.3	B-other	AALL0232	Phase 2
TARGET-10-PARCHU-09A-01D	Male	B-ALL	2.6	Hyperdiploidy	AALL0331	Phase 2
TARGET-10-PARCHY-09A-01D	Male	B-ALL	2.9	Hyperdiploidy	AALL0331	Phase 2
TARGET-10-PARCKD-09A-01D	Male	B-ALL	2.5	B-other	AALL0331	Phase 2
TARGET-10-PARCKJ-03A-01D	Female	B-ALL	2.3	Hyperdiploidy	AALL0331	Phase 2
TARGET-10-PARCLU-09A-01D	Female	B-ALL	16.6	B-other	AALL0232	Phase 2
TARGET-10-PARCLW-09A-01D	Female	B-ALL	3.3	*ETV6-RUNX1*	AALL0331	Phase 2
TARGET-10-PARCMD-03A-01D	Female	B-ALL	1.7	Hyperdiploidy	AALL0331	Phase 2
TARGET-10-PARCMG-03A-01D	Male	B-ALL	4.0	*ETV6-RUNX1*	AALL0232	Phase 2
TARGET-10-PARCSH-09A-01D	Female	B-ALL	10.6	*BCR-ABL1*	AALL0232	Phase 2
TARGET-10-PARCTN-09A-01D	Female	B-ALL	10.7	*TCF3-PBX1*	AALL0232	Phase 2
TARGET-10-PARCUM-09A-01D	Male	B-ALL	11.1	B-other	AALL0232	Phase 2
TARGET-10-PARCUW-09A-01D	Female	B-ALL	3.7	*ETV6-RUNX1*	AALL0331	Phase 2
TARGET-10-PARCVB-09A-01D	Female	B-ALL	2.7	B-other	AALL0331	Phase 2
TARGET-10-PARCVT-09A-01D	Female	B-ALL	14.8	Hypodiploidy	AALL0232	Phase 2
TARGET-10-PARCWB-09A-01D	Male	B-ALL	4.5	B-other	AALL0331	Phase 2
TARGET-10-PARCZY-09A-01D	Male	B-ALL	1.7	*ETV6-RUNX1*	AALL0232	Phase 2
TARGET-10-PARDAK-09A-01D	Male	B-ALL	8.5	KMT2Ar	AALL0232	Phase 2
TARGET-10-PARDBN-09A-01D	Male	B-ALL	15.9	B-other	AALL0232	Phase 2
TARGET-10-PARDBT-09A-01D	Female	B-ALL	4.2	*ETV6-RUNX1*	AALL0232	Phase 2
TARGET-10-PARDCJ-09A-01D	Female	B-ALL	16.1	B-other	AALL0232	Phase 2
TARGET-10-PARDCR-09A-01D	Male	B-ALL	4.5	*ETV6-RUNX1*	AALL0331	Phase 2
TARGET-10-PARDDM-09A-01D	Male	B-ALL	4.8	Hyperdiploidy	AALL0331	Phase 2
TARGET-10-PARDDV-09A-01D	Male	B-ALL	3.3	*ETV6-RUNX1*	AALL0331	Phase 2
TARGET-10-PARDDW-09A-01D	Male	B-ALL	4.5	B-other	AALL0331	Phase 2

TARGET-10-PARDEJ-09A-01D	Male	B-ALL	3.4	B-other	AALL0232	Phase 2
TARGET-10-PARDEY-09A-01D	Female	B-ALL	5.6	*ETV6-RUNX1*	AALL0331	Phase 2
TARGET-10-PARDFB-09A-01D	Male	B-ALL	3.1	B-other	AALL0232	Phase 2
TARGET-10-PARDFH-09A-01D	Female	B-ALL	4.2	*ETV6-RUNX1*	AALL0331	Phase 2
TARGET-10-PARDFI-09A-01D	Female	B-ALL	3.1	*ETV6-RUNX1*	AALL0331	Phase 2
TARGET-10-PARDFN-09A-01D	Male	B-ALL	10.9	Hyperdiploidy	AALL0232	Phase 2
TARGET-10-PARDHK-09A-01D	Female	B-ALL	1.8	*ETV6-RUNX1*	AALL0331	Phase 2
TARGET-10-PARDHW-09A-01D	Female	B-ALL	1.4	Hyperdiploidy	AALL0331	Phase 2
TARGET-10-PARDIN-09A-01D	Male	B-ALL	3.4	Hyperdiploidy	AALL0331	Phase 2
TARGET-10-PARDKG-09A-01D	Male	B-ALL	9.8	B-other	AALL0232	Phase 2
TARGET-10-PARDLJ-09A-01D	Male	B-ALL	14.9	B-other	AALL0232	Phase 2
TARGET-10-PARDLP-09A-01D	Female	B-ALL	4.4	Hyperdiploidy	AALL0331	Phase 2
TARGET-10-PARDLR-09A-01D	Female	B-ALL	8.3	Hyperdiploidy	AALL0331	Phase 2
TARGET-10-PARDLZ-09A-01D	Male	B-ALL	11.5	*ETV6-RUNX1*	AALL0232	Phase 2
TARGET-10-PARDMI-09A-01D	Female	B-ALL	4.2	*ETV6-RUNX1*	AALL0331	Phase 2
TARGET-10-PARDRI-09A-01D	Male	B-ALL	9.9	*ETV6-RUNX1*	AALL0331	Phase 2
TARGET-10-PARDRS-09A-01D	Female	B-ALL	8.7	Hyperdiploidy	AALL0331	Phase 2
TARGET-10-PARDSN-09A-01D	Male	B-ALL	6.1	Hyperdiploidy	AALL0331	Phase 2
TARGET-10-PARDSP-09A-01D	Female	B-ALL	5.9	Hyperdiploidy	AALL0331	Phase 2
TARGET-10-PARDST-09A-01D	Male	B-ALL	5.5	*ETV6-RUNX1*	AALL0232	Phase 2
TARGET-10-PARDUM-09A-01D	Female	B-ALL	5.8	Hyperdiploidy	AALL0232	Phase 2
TARGET-10-PARDVD-09A-01D	Female	B-ALL	11.5	*ETV6-RUNX1*	AALL0232	Phase 2
TARGET-10-PARDWM-09A-01D	Female	B-ALL	15.5	B-other	AALL0232	Phase 2
TARGET-10-PARDXG-09A-01D	Female	B-ALL	12.9	Hyperdiploidy and *ETV6-RUNX1*	AALL0232	Phase 2
TARGET-10-PARDXI-03A-01D	Male	B-ALL	10.4	Hyperdiploidy	AALL0232	Phase 2
TARGET-10-PARDXS-09A-01D	Male	B-ALL	3.7	Hyperdiploidy	AALL0232	Phase 2
TARGET-10-PAREAA-09A-01D	Female	B-ALL	5.1	B-other	AALL0232	Phase 2
TARGET-10-PAREAL-09A-01D	Female	B-ALL	9.3	*TCF3-PBX1*	AALL0331	Phase 2
TARGET-10-PAREBA-09A-01D	Male	B-ALL	2.9	Hyperdiploidy	AALL0331	Phase 2
TARGET-10-PAREDS-03A-01D	Male	B-ALL	1.5	B-other	AALL0331	Phase 2
TARGET-10-PAREEE-09A-01D	Male	B-ALL	4.8	Hyperdiploidy	AALL0331	Phase 2
TARGET-10-PAREEX-09A-01D	Female	B-ALL	7.7	Hyperdiploidy	AALL0331	Phase 2
TARGET-10-PAREGE-09A-01D	Male	B-ALL	6.7	B-other	AALL0331	Phase 2
TARGET-10-PAREHN-09A-01D	Male	B-ALL	15.5	B-other	AALL0232	Phase 2
TARGET-10-PAREIN-09A-01D	Male	B-ALL	3.6	*ETV6-RUNX1*	AALL0331	Phase 2
TARGET-10-PAREIV-09A-01D	Male	B-ALL	3.0	B-other	AALL0331	Phase 2
TARGET-10-PAREJA-09A-01D	Female	B-ALL	7.1	Hyperdiploidy	AALL0331	Phase 2
TARGET-10-PAREJZ-09A-01D	Male	B-ALL	16.2	B-other	AALL0232	Phase 2
TARGET-10-PAREKG-09A-01D	Female	B-ALL	8.1	iAMP21	AALL0232	Phase 2
TARGET-10-PAREKH-09A-01D	Male	B-ALL	5.3	Hyperdiploidy	AALL0331	Phase 2
TARGET-10-PAREKM-09A-01D	Male	B-ALL	4.3	Hyperdiploidy	AALL0331	Phase 2
TARGET-10-PARENT-09A-01D	Male	B-ALL	2.8	B-other	AALL0331	Phase 2
TARGET-10-PARENU-09A-01D	Female	B-ALL	1.9	Hyperdiploidy	AALL0331	Phase 2
TARGET-10-PAREPB-09A-01D	Male	B-ALL	3.4	Hyperdiploidy	AALL0331	Phase 2
TARGET-10-PAREPF-09A-01D	Male	B-ALL	5.5	*ETV6-RUNX1*	AALL0331	Phase 2
TARGET-10-PARERM-09A-01D	Female	B-ALL	3.5	B-other	AALL0232	Phase 2
TARGET-10-PARERS-09A-01D	Female	B-ALL	4.0	Hyperdiploidy	AALL0331	Phase 2
TARGET-10-PARESP-09A-01D	Male	B-ALL	1.6	B-other	AALL0232	Phase 2

TARGET-10-PAREST-09A-01D	Female	B-ALL	3.8	Hyperdiploidy	AALL0331	Phase 2
TARGET-10-PARETC-09A-01D	Female	B-ALL	5.6	iAMP21	AALL0331	Phase 2
TARGET-10-PARETH-09A-01D	Male	B-ALL	7.4	B-other	AALL0232	Phase 2
TARGET-10-PARETV-09A-01D	Female	B-ALL	8.0	Hyperdiploidy	AALL0331	Phase 2
TARGET-10-PAREUH-09A-01D	Female	B-ALL	7.4	*ETV6-RUNX1*	AALL0331	Phase 2
TARGET-10-PAREWM-09A-01D	Male	B-ALL	6.8	B-other	AALL0331	Phase 2
TARGET-10-PAREYJ-09A-01D	Male	B-ALL	4.8	*ETV6-RUNX1*	AALL0331	Phase 2
TARGET-10-PAREYW-09A-01D	Male	B-ALL	17.2	B-other	AALL0232	Phase 2
TARGET-10-PARFAP-09A-01D	Male	B-ALL	5.6	*ETV6-RUNX1*	AALL0331	Phase 2
TARGET-10-PARFDB-09A-01D	Male	B-ALL	14.8	B-other	AALL0232	Phase 2
TARGET-10-PARFDG-09A-01D	Male	B-ALL	7.6	Hyperdiploidy	AALL0331	Phase 2
TARGET-10-PARFDW-09A-01D	Male	B-ALL	3.7	*ETV6-RUNX1*	AALL0331	Phase 2
TARGET-10-PARFEH-09A-01D	Male	B-ALL	2.2	Hyperdiploidy	AALL0232	Phase 2
TARGET-10-PARFFC-03A-01D	Male	B-ALL	2.2	*BCR-ABL1*	AALL0331	Phase 2
TARGET-10-PARFHH-09A-01D	Male	B-ALL	5.7	*ETV6-RUNX1*	AALL0331	Phase 2
TARGET-10-PARFIZ-09A-01D	Female	B-ALL	7.2	Hyperdiploidy	AALL0331	Phase 2
TARGET-10-PARFJK-09A-01D	Female	B-ALL	3.4	Hyperdiploidy	AALL0331	Phase 2
TARGET-10-PARFJM-09A-01D	Male	B-ALL	3.5	Hyperdiploidy	AALL0331	Phase 2
TARGET-10-PARGBK-09A-01D	Male	B-ALL	13.3	Hyperdiploidy	AALL0232	Phase 2
TARGET-10-PARGBR-09A-01D	Male	B-ALL	17.6	B-other	AALL0232	Phase 2
TARGET-10-PARGBT-09A-01D	Female	B-ALL	10.6	B-other	AALL0232	Phase 2
TARGET-10-PARGGI-09A-01D	Female	B-ALL	10.2	B-other	AALL0232	Phase 2
TARGET-10-PARGJY-09A-01D	Female	B-ALL	17.8	B-other	AALL0232	Phase 2
TARGET-10-PARGKD-09A-01D	Female	B-ALL	4.2	*ETV6-RUNX1*	AALL0232	Phase 2
TARGET-10-PARGLE-09A-01D	Male	B-ALL	11.5	*BCR-ABL1*	AALL0232	Phase 2
TARGET-10-PARGLI-09A-01D	Male	B-ALL	12.5	B-other	AALL0232	Phase 2
TARGET-10-PARGLW-09A-01D	Male	B-ALL	10.3	B-other	AALL0232	Phase 2
TARGET-10-PARGYV-09A-01D	Male	B-ALL	4.2	*BCR-ABL1*	AALL0232	Phase 2
TARGET-10-PARHAN-09A-01D	Female	B-ALL	15.0	B-other	AALL0232	Phase 2
TARGET-10-PARHFF-09A-01D	Female	B-ALL	10.2	Hyperdiploidy	AALL0232	Phase 2
TARGET-10-PARHLM-09A-01D	Female	B-ALL	2.2	Hyperdiploidy	AALL0232	Phase 2
TARGET-10-PARHMT-09A-01D	Male	B-ALL	14.9	B-other	AALL0232	Phase 2
TARGET-10-PARHSD-09A-01D	Female	B-ALL	3.9	Hyperdiploidy	AALL0232	Phase 2
TARGET-10-PARIBB-09A-01D	Female	B-ALL	11.7	Hyperdiploidy	AALL0232	Phase 2
TARGET-10-PARIIA-09A-01D	Female	B-ALL	13.2	B-other	AALL0232	Phase 2
TARGET-10-PARJBB-09A-01D	Male	B-ALL	2.2	B-other	AALL0232	Phase 2
TARGET-10-PARJBZ-03A-01D	Male	B-ALL	14.0	Hyperdiploidy	AALL0232	Phase 2
TARGET-10-PARJJP-09A-01D	Male	B-ALL	12.8	B-other	AALL0232	Phase 2
TARGET-10-PARJMY-09A-01D	Female	B-ALL	12.9	B-other	AALL0232	Phase 2
TARGET-10-PARJNL-09A-01D	Female	B-ALL	10.0	B-other	AALL0232	Phase 2
TARGET-10-PARJNR-09A-01D	Female	B-ALL	11.0	B-other	AALL0232	Phase 2
TARGET-10-PARJRT-09A-01D	Female	B-ALL	2.4	B-other	AALL0232	Phase 2
TARGET-10-PARJWB-09A-01D	Female	B-ALL	13.5	B-other	AALL0232	Phase 2
TARGET-10-PARJYV-09A-01D	Male	B-ALL	11.9	B-other	AALL0232	Phase 2
TARGET-10-PARKBT-09A-01D	Male	B-ALL	15.7	Hyperdiploidy	AALL0232	Phase 2
TARGET-10-PARKEN-03A-01D	Male	B-ALL	1.7	Hyperdiploidy	AALL0232	Phase 2
TARGET-10-PARKEU-09A-01D	Male	B-ALL	5.7	Hyperdiploidy	AALL0232	Phase 2
TARGET-10-PARKFN-09A-01D	Female	B-ALL	3.0	B-other	AALL0232	Phase 2
TARGET-10-PARKFU-09A-01D	Male	B-ALL	15.1	B-other	AALL0232	Phase 2
TARGET-10-PARLBP-09A-01D	Male	B-ALL	10.0	B-other	AALL0232	Phase 2

TARGET-10-PARLDF-09A-01D	Male	B-ALL	4.1	*ETV6-RUNX1*	AALL0232	Phase 2
TARGET-10-PARLFI-09A-01D	Male	B-ALL	14.0	KMT2Ar	AALL0232	Phase 2
TARGET-10-PARLMI-09A-01D	Female	B-ALL	1.3	Hyperdiploidy	AALL0232	Phase 2
TARGET-10-PARLPB-09A-01D	Male	B-ALL	14.4	B-other	AALL0232	Phase 2
TARGET-10-PARLTX-09A-01D	Male	B-ALL	1.6	Hyperdiploidy	AALL0232	Phase 2
TARGET-10-PARMMA-09A-01D	Female	B-ALL	5.1	*ETV6-RUNX1*	AALL0232	Phase 2
TARGET-10-PARMSB-09A-01D	Female	B-ALL	13.9	B-other	AALL0232	Phase 2
TARGET-10-PARMYP-09A-01D	Male	B-ALL	5.7	Hyperdiploidy	AALL0232	Phase 2
TARGET-10-PARNDB-09A-01D	Female	B-ALL	10.1	*TCF3-PBX1*	AALL0232	Phase 2
TARGET-10-PARNGI-09A-01D	Male	B-ALL	2.7	B-other	AALL0232	Phase 2
TARGET-10-PARNIZ-09A-01D	Male	B-ALL	1.7	B-other	AALL0232	Phase 2
TARGET-10-PARNSW-09A-01D	Male	B-ALL	13.7	Hyperdiploidy and *BCR-ABL1*	AALL0232	Phase 2
TARGET-10-PARPDP-09A-01D	Male	B-ALL	16.9	KMT2Ar	AALL0232	Phase 2
TARGET-10-PARPFF-09A-01D	Female	B-ALL	15.5	B-other	AALL0232	Phase 2
TARGET-10-PARPGJ-09A-01D	Male	B-ALL	11.3	iAMP21 and *ETV6-RUNX1*	AALL0232	Phase 2
TARGET-10-PARPGL-09A-01D	Female	B-ALL	13.0	*ETV6-RUNX1*	AALL0232	Phase 2
TARGET-10-PARPGW-09A-01D	Female	B-ALL	4.4	*ETV6-RUNX1*	AALL0232	Phase 2
TARGET-10-PARPPV-09A-01D	Male	B-ALL	14.1	*TCF3-PBX1*	AALL0232	Phase 2
TARGET-10-PARPXV-09A-01D	Male	B-ALL	4.4	*ETV6-RUNX1*	AALL0232	Phase 2
TARGET-10-PARPYH-09A-01D	Male	B-ALL	5.8	*ETV6-RUNX1*	AALL0232	Phase 2
TARGET-10-PARPYS-09A-01D	Male	B-ALL	14.7	B-other	AALL0232	Phase 2
TARGET-10-PARPZI-09A-01D	Male	B-ALL	6.3	B-other	AALL0232	Phase 2
TARGET-10-PARPZR-09A-01D	Female	B-ALL	1.3	KMT2Ar	AALL0232	Phase 2
TARGET-10-PARRKG-03A-01D	Male	B-ALL	7.2	B-other	AALL0232	Phase 2
TARGET-10-PARRMU-09A-01D	Female	B-ALL	14.2	Hyperdiploidy	AALL0232	Phase 2
TARGET-10-PARRSR-09A-01D	Female	B-ALL	13.7	B-other	AALL0232	Phase 2
TARGET-10-PARRVK-09A-01D	Female	B-ALL	17.6	B-other	AALL0232	Phase 2
TARGET-10-PARRYW-09A-01D	Male	B-ALL	11.9	Hyperdiploidy	AALL0232	Phase 2
TARGET-10-PARSGM-09A-01D	Male	B-ALL	13.3	Hyperdiploidy	AALL0232	Phase 2
TARGET-10-PARSHV-09A-01D	Male	B-ALL	13.9	B-other	AALL0232	Phase 2
TARGET-10-PARSJH-09A-01D	Male	B-ALL	16.7	*BCR-ABL1*	AALL0232	Phase 2
TARGET-10-PARSKH-09A-01D	Female	B-ALL	14.9	Hypodiploidy	AALL0232	Phase 2
TARGET-10-PARSKV-09A-01D	Male	B-ALL	7.0	B-other	AALL0232	Phase 2
TARGET-10-PARSKY-09A-01D	Male	B-ALL	3.4	Hyperdiploidy	AALL0232	Phase 2
TARGET-10-PARSRI-09A-01D	Female	B-ALL	15.5	B-other	AALL0232	Phase 2
TARGET-10-PARSSV-09A-01D	Female	B-ALL	6.8	B-other	AALL0232	Phase 2
TARGET-10-PARSTB-09A-01D	Male	B-ALL	15.4	B-other	AALL0232	Phase 2
TARGET-10-PARTAK-09A-01D	Male	B-ALL	10.7	B-other	AALL0232	Phase 2
TARGET-10-PARTEF-09A-01D	Female	B-ALL	3.1	*ETV6-RUNX1*	AALL0232	Phase 2
TARGET-10-PARTGW-09A-01D	Female	B-ALL	5.1	*TCF3-PBX1*	AALL0232	Phase 2
TARGET-10-PARTID-09A-01D	Female	B-ALL	14.6	B-other	AALL0232	Phase 2
TARGET-10-PARTIK-09A-01D	Male	B-ALL	3.8	*ETV6-RUNX1*	AALL0232	Phase 2
TARGET-10-PARTJJ-09A-01D	Male	B-ALL	3.2	*ETV6-RUNX1*	AALL0232	Phase 2
TARGET-10-PARTSC-09A-01D	Male	B-ALL	8.3	*ETV6-RUNX1*	AALL0232	Phase 2
TARGET-10-PARTWH-09A-01D	Male	B-ALL	11.7	B-other	AALL0232	Phase 2
TARGET-10-PARTZE-09A-01D	Male	B-ALL	12.5	*TCF3-PBX1*	AALL0232	Phase 2
TARGET-10-PARTZJ-09A-01D	Male	B-ALL	2.9	*ETV6-RUNX1*	AALL0232	Phase 2
TARGET-10-PARUBN-09A-01D	Female	B-ALL	12.6	B-other	AALL0232	Phase 2

TARGET-10-PARUBU-09A-01D	Male	B-ALL	3.1	*ETV6-RUNX1*	AALL0232	Phase 2
TARGET-10-PARUBX-09A-01D	Male	B-ALL	10.3	B-other	AALL0232	Phase 2
TARGET-10-PARUCI-09A-01D	Female	B-ALL	3.3	Hyperdiploidy	AALL0232	Phase 2
TARGET-10-PARUCT-09A-01D	Male	B-ALL	14.7	*ETV6-RUNX1*	AALL0232	Phase 2
TARGET-10-PARUGV-09A-01D	Female	B-ALL	1.6	*ETV6-RUNX1*	AALL0232	Phase 2
TARGET-10-PARUIT-09A-01D	Male	B-ALL	1.1	KMT2Ar	AALL0232	Phase 2
TARGET-10-PARUKK-09A-01D	Male	B-ALL	15.5	B-other	AALL0232	Phase 2
TARGET-10-PARURK-09A-01D	Female	B-ALL	13.5	Hyperdiploidy	AALL0232	Phase 2
TARGET-10-PARUYE-03A-01D	Female	B-ALL	1.7	B-other	AALL0232	Phase 2
TARGET-10-PARUYH-09A-01D	Female	B-ALL	3.3	*ETV6-RUNX1*	AALL0232	Phase 2
TARGET-10-PARUYU-09A-01D	Male	B-ALL	11.0	B-other	AALL0232	Phase 2
TARGET-10-PARVAK-09A-01D	Female	B-ALL	6.0	*TCF3-PBX1*	AALL0232	Phase 2
TARGET-10-PARVBL-09A-01D	Female	B-ALL	11.2	B-other	AALL0232	Phase 2
TARGET-10-PARVBY-09A-01D	Male	B-ALL	2.7	Hyperdiploidy	AALL0232	Phase 2
TARGET-10-PARVCG-09A-01D	Female	B-ALL	10.0	*ETV6-RUNX1*	AALL0232	Phase 2

* Determined by karyotyping (G-banding), DNA-index measurement and fluorescence *in situ* hybridization
** Patients negative for hyperdiploidy, hypodiploidy, *ETV6-RUNX1* fusion, *BCR-ABL1* fusion, iAMP21, *KMT2A*r and *TCF3-PBX1* fusion
*** At least 5 copies of *RUNX1* gene detected

Table S4. Distribution of genetic subtypes in our in-house discovery patient cohort and in the merged TARGET ALL Phase 1 Pilot and Phase 2 Expansion study cohort used for validation.

**Genetic subtype**	**Discovery cohort** ***N = 260***	**TARGET ALL P1 & P2 validation cohort** ***N = 606***
Hyperdiploidy	31.9%	24.1%
ETV6-RUNX1	26.2%	19.5%
BCR-ABL1	2.3%	1.0%
iAMP21	3.8%	1.8%
KMT2Ar	1.5%	4.1%
TCF3-PBX1	1.2%	6.1%
B-other	34.2%	42.9%

Table S5. Distribution of numerical and subchromosomal copy number aberrations across genetic disease subtypes in our patient cohort.

Numerical chromosomal aberrations							
**Genetic subtype**	**Sum of CNAs**	**CNAs per patient**	**Copy number gains**	**Copy number losses**	**Multiple gains***	**Biallelic losses***	**Subclonal CNAs***
Hyperdiploidy	680	8.19	676	7	139	1	6
ETV6-RUNX1	33	0.48	22	11	2	0	2
BCR-ABL1	7	1.17	5	0	2	0	0
iAMP21	2	0.20	2	0	0	0	0
KMT2Ar	0	0.00	0	0	0	0	0
TCF3-PBX1	0	0.00	0	0	0	0	0
B-other	55	0.62	41	11	6	0	1
All samples**	761	2.93	734	27	147	1	8

Subchromosomal aberrations					
**Genetic subtype**	**Sum of CNAs**	**CNAs per patient**	**Copy number gains**	**Copy number losses**	**Subclonal CNAs***
Hyperdiploidy	96	1.16	38	57	14
ETV6-RUNX1	259	3.81	41	218	28
BCR-ABL1	29	4.83	4	25	4
iAMP21	50	5.00	21	29	1
KMT2Ar	13	3.25	0	13	1
TCF3-PBX1	5	1.67	2	3	0
B-other	199	2.24	29	170	17
All samples**	637	2.46	131	508	61

* Values represent a subset of copy number gains, copy number losses or sum of CNAs
** Values are not the sum of subtype-specific values due to three patients who showed features of two different subtypes

Table S6. Prognostic influence of genetic aberrations assessed by univariate Cox regression models.

Genetic aberration*	Hazard ratio	Score
IKZF1 loss	4.82E+00	-4
BCR-ABL1 fusion	4.60E+00	-4
PTEN loss	4.49E+00	-4
P2RY8-CRLF2 fusion	4.30E+00	-4
KMT2A rearrangement	4.20E+00	-4
LEF1 loss	4.10E+00	-4
iAMP21	2.50E+00	-2
HHD without +4 and +6	2.30E+00	-2
TP53 loss	2.00E+00	-2
MLLT3 loss	1.90E+00	-1
B-other	1.80E+00	-1
CDKN2A/B loss	1.51E+00	-1
ERG gain	1.43E+00	0
PAX5 loss	1.40E+00	0
PAR1 loss	1.40E+00	0
RUNX1 gain	1.20E+00	0
PDGFRB loss	1.17E+00	0
JAK2 loss	1.10E+00	0
MTAP loss	1.10E+00	0
BTG1 loss	1.10E+00	0
CD200/BTLA loss	1.10E+00	0
EBF1 loss	9.40E-01	0
terminal 5q loss	9.20E-01	0
NR3C1 loss	6.90E-01	0
VPREB1 loss	6.90E-01	0
RB1 loss	6.00E-01	+1
CTCF loss	5.90E-01	+1
ETV6 loss	3.00E-01	+2
HHD with +4 and +6	2.40E-01	+4
ETV6-RUNX1 fusion	2.40E-01	+4
TBL1XR1 loss	2.40E-01	+4

ERG loss	1.13E-07	+4
RAG2 loss	3.90E-08	+4
PHF6 gain	3.82E-08	+4
CASP8AP2 loss	3.70E-08	+4

* Aberrations with <1.5% abundance were excluded. Scores were assigned based on hazard ratio ranges: 0.00-0.25: +4, 0.26-0.33: +3, 0.34-0.50: +2, 0.51-0.66: +1, 1.51-2.00: -1, 2.01-3.00: -2, 3.01-4.00: -3, >4.01: -4
